# Deubiquitinase USP32 stabilizes DDX17 to promote tumor progression and lenvatinib resistance in hepatocellular carcinoma via the miR-3163/MAPK pathway

**DOI:** 10.1038/s41419-026-09040-1

**Published:** 2026-06-25

**Authors:** Yang Yu, Bo-Rui Xu, Hao-Ran Huang, Zi-Wen Nong, Fei-Feng Wu, Guo-Pei Zhang, Shao-Qiang Li

**Affiliations:** Department of Liver Surgery, Center of Hepato-Pancreato-Biliary Surgery, The First Affiliated Hospital of Sun Yat-sen University, Guangzhou, 510080 China

**Keywords:** Oncogenes, Cancer therapeutic resistance

## Abstract

Lenvatinib, a first-line therapy for hepatocellular carcinoma (HCC), is frequently compromised by the development of acquired resistance. Although aberrant protein ubiquitination contributes to drug resistance, the underlying mechanisms remain incompletely understood. In this study, we identify ubiquitin-specific protease 32 (USP32) as a clinically significant oncogene that is overexpressed in HCC and closely associated with aggressive tumor progression and lenvatinib resistance. USP32 knockdown markedly inhibits tumor growth, metastasis, and restores lenvatinib sensitivity both in vitro and in vivo, whereas USP32 overexpression aggravates malignant behaviors. Mechanistically, USP32 directly interacts with and deubiquitinates DEAD-box helicase 17 (DDX17) by cleaving K48-linked polyubiquitin chains, thereby stabilizing DDX17. Furthermore, DDX17 transcriptionally represses the tumor-suppressive microRNA miR-3163, leading to hyperactivation of the MAPK signaling pathway involved in lenvatinib resistance. Notably, combined treatment with the MAPK inhibitor selumetinib and lenvatinib synergistically overcomes drug resistance in lenvatinib-resistant xenograft models. Collectively, our findings identify USP32 as an important regulator of HCC malignancy and lenvatinib resistance via the DDX17/miR-3163/MAPK axis, highlighting its potential as a therapeutic target in refractory HCC.

## Introduction

Hepatocellular carcinoma (HCC) remains one of the most lethal malignancies worldwide, ranking as the fourth leading cause of cancer-related mortality [1]. Notably, more than 50% of HCC patients are diagnosed at an advanced, unresectable stage [2–4]. Lenvatinib, a tyrosine kinase inhibitor (TKI), has been approved as the first-line therapy for inoperable HCC by treatment guidelines worldwide [5–7]. However, the emergence of acquired drug resistance significantly limits the clinical efficacy of lenvatinib [8, 9]. Therefore, there is an urgent need to elucidate the underlying mechanisms of driving HCC progression and lenvatinib resistance in order to identify novel and effective therapeutic targets.

Recent studies underscore that the ubiquitin-proteasome system (UPS) plays a pivotal role in cellular homeostasis by regulating protein degradation and function. Deubiquitinating enzymes (DUBs), as key components of the UPS, reverse ubiquitination to regulate the stability, activity, and localization of substrate proteins. Dysregulation of DUBs has been reported across multiple malignancies, impacting key signaling pathways that promote tumor growth, metastasis, and drug resistance [10–15]. Among these, ubiquitin-specific protease 32 (USP32) is demonstrated to promote proliferation, invasion, and metastasis in non-small cell lung cancer [16]. Additionally, USP32 drives gastric cancer progression and confers resistance to cisplatin [17]. However, its involvement in HCC progression and drug resistance has yet to be fully elucidated.

DDX17, a multifunctional RNA helicase of the DEAD-box family, has been reported to regulate RNA metabolism, alternative splicing, and gene transcription [18]. Emerging evidence highlights DDX17 as a critical mediator in driving oncogenic processes. For instance, studies indicate that DDX17 drives epithelial-mesenchymal transition (EMT) and metastasis in colorectal cancer cells [19]. Furthermore, DDX17 promotes HCC progression and confers resistance to sorafenib [20]. Although DDX17 has been implicated in various malignancies, the post-translational regulation of DDX17 by ubiquitination and deubiquitination, as well as the mechanisms of its oncogenic role in HCC remain insufficiently understood.

This study delineates a novel oncogenic regulatory axis in HCC, comprising USP32, DDX17, and miRNA-3163, which converges on MAPK signaling to drive tumor progression and lenvatinib resistance. By integrating molecular, cellular, and preclinical models, we aim to uncover actionable insights into this pathway and propose USP32 or its downstream effectors as potential therapeutic targets. Our findings have the potential to guide the development of next-generation combination therapies that could overcome lenvatinib resistance and improve clinical outcomes for HCC patients. Understanding these interactions will provide novel insights into the molecular pathogenesis of HCC and open avenues for overcoming therapeutic resistance, ultimately improving clinical outcomes for patients.

## Methods

### Cell culture

The human HCC cell lines SNU-449, Hep3B, HepG2, HCCLM3, PLC/PRF/5, MHCC-97H, Huh7 and human embryonic kidney HEK293T cells were obtained from Shanghai Cell Bank (Chinese Academy of Science, Shanghai, China). SNU-449, Hep3B, HepG2, HCCLM3, PLC/PRF/5, MHCC-97H, Huh7 and HEK293T cells were cultured in Dulbecco’s modified Eagle’s medium (DMEM, C11995500BT, Gibco) supplemented with 10% fetal bovine serum (FBS, Procell, 164210-50). All cells were maintained at 37 °C in a humidified incubator with 5% CO_2_.

### Subcutaneous tumor and lung metastasis model of HCC

For the subcutaneous tumor model, 1 × 10^6^ HCC cells were resuspended in 100 μL PBS and injected subcutaneously into the flanks of BALB/c nude mice aged 6 weeks. Under pre-set instruments, we used a vernier caliper to measure the tumor sizes and body weight every two days until the end of the experiment or tumors reached ethical endpoints, or if mice showed signs of ill health, and tumors were excised for histological and molecular analyses. For the lung metastasis model, 3 × 10^6^ HCC cells in 0.2 mL of PBS were injected into the tail vein of each mouse. Four weeks later, lung ex vivo imaging was performed to examine tumor metastasis. Then mice were euthanized, and lung tissues were harvested for subsequent tests. Animal experiments were conducted according to the protocols approved by the Ethics Committee of the First Affiliated Hospital of Sun Yat-sen University (Approval No [2024]059).

### Patient cohorts and clinical samples

This study utilized two independent patient cohorts. Cohort 1 retrospectively enrolled patients with HCC who underwent curative resection at the First Affiliated Hospital of Sun Yat-sen University between January 2022 and October 2024. HCC tissue specimens were used to analyze the correlation between USP32 expression and clinicopathological characteristics as well as patient prognosis. Comprehensive clinical data were obtained, including age, gender, liver function parameters, tumor stage, tumor size and number, differentiation grade, and histopathological slides. Cohort 2 consisted of 8 matched pairs of fresh HCC and adjacent non-tumorous liver tissues from patients who underwent surgical resection between October 2022 and February 2023. These samples were prospectively collected for the specific purpose of quantifying USP32 mRNA and protein expression levels in tumor versus peri-tumoral tissues.

The collection and use of clinical data and tissue samples complied with the principles of the Declaration of Helsinki. The study protocol was approved by the Institutional Review Board and Ethics Committee of the First Affiliated Hospital of Sun Yat-sen University (Approval No [2024]339). Written informed consent was obtained from each participating patient.

### Plasmids and RNA inference

We obtained the wild-type/mutant constructs of USP32, DDX17, and hsa-miR-3163 from Genecreate (Wuhan, China). Full length and indicated truncated mutation of USP32 were cloned into the pcDNA3.1-Flag. Full length and indicated truncated mutation of DDX17 were cloned into the pcDNA3.1-Myc. The HA-Ub, -K48, and -K63 plasmids were obtained from HanYi Biosciences Inc (Guangzhou, China). We transfected the plasmids into cells using Lipofectamine 3000 (L3000015, Invitrogen). Small interfering RNAs (siRNAs) and short hairpin RNAs (shRNAs) were used for specific gene knockdown. The siRNA and shRNA sequences targeting USP32 and DDX17 are listed in Table S1.

### Quantitative real-time reverse transcription PCR

RNA-QUICK Purification kit (ES Scicence, Shanghai, China) was used to isolate the total RNA, and Reverse transcription was performed using the ABScript Neo RT Master Mix for qPCR with gDNA Remover (RK20433, ABclonal, Wuhan, China) according to the instructions. AFTSpin Tissue/Cell Fast miRNA Extraction Kit (RK30126, ABclonal, Wuhan, China) was used to isolate the miRNA from the cancer cells and reverse transcription. BrightCycl-e Universal SYBR Green qPCR Mix with UDG (RK21219, ABclonal, Wuhan, China) for total RNA, 2X Universal SYBR Green FastqPCR Mix (RK21203, ABclonal, Wuhan, China) for miRNA, and 7500 Fast Real-Time PCR System (Applied Biosystems, Singapore) were used for qRT-PCR analysis. The primer sequences used for mRNA and miRNA detection are listed in Table S1.

### Wound healing assay

To assess cell migration, a wound healing assay was conducted as follows. Cells were plated onto 6-well plates and grown until they reached confluence. A straight line was then scratched into the cell monolayer using a 200-μL pipette tip. After removing non-adherent cells by gentle washing, fresh medium was added to the wells. Cell migration into the wounded area was monitored by capturing images at 0, 12, 24, and 36 h using a phase-contrast microscope. The migration distance was calculated by measuring the reduction in the wound width over time.

### Colony formation assay

Cells were harvested, counted, and plated at a density of 1000 cells per 60-mm dish. The dishes were maintained in a humidified incubator at 37°C with 5% CO2 for approximately two weeks. Medium changes were performed every 2-3 days to prevent cell death due to nutrient depletion. Following the incubation period, the colonies were fixed with 4% paraformaldehyde and stained with 0.1% crystal violet. Colonies containing more than 50 cells were counted under a dissecting microscope. The plating efficiency was determined by dividing the number of colonies counted by the number of cells plated and expressed as a percentage.

### Cell counting Kit-8 assay

Cells were first trypsinized and counted. Subsequently, they were plated into 96-well plates at a density of 8000 cells per well in 100 μL of complete medium. After a 24-h incubation period to allow cell attachment, the medium was replaced with fresh medium containing different concentrations of the test compound. A control group was included, which received only medium without the test compound. The plates were then incubated at 37°C with 5% CO2 for the specified time. Following incubation, 10 μL of CCK-8 reagent was added to each well, and the plates were incubated for an additional 2 h. The absorbance at 450 nm was determined using a spectrophotometer, and cell viability was calculated based on the absorbance values.

### Transwell assay

We performed transwell invasion assay using 8 μm pore polycarbonate membrane transwell plates (Corning, USA). Briefly, 1 × 10^6^ cells were suspended without serum and were cultured in the upper compartment of a transwell insert precoated with or without Matrigel for 2 h at 37°C. The lower chambers were filled with 600 μl complete medium supplemented with 20% fetal bovine serum. The assembly was then placed in a humidified incubator at 37°C with 5% CO2. After 24-72 h, the invasion cells were fixed and visualized by crystal violet staining.

### Western blot analysis

For Western blot analysis, protein lysates were first prepared, and the concentrations were determined via BCA protein assay. SDS-PAGE was employed to resolve protein fractions, which were then electrophoretically transferred to polyvinylidene difluoride (PVDF) membranes. Before antibody probing, membranes were blocked with 5% non-fat milk or bovine serum albumin (BSA) in TBST for 2 h at room temperature. Primary antibodies targeting the proteins of interest were incubated with the membranes at 4°C overnight. Following extensive washing, membranes were incubated with appropriate HRP-labeled secondary antibodies for 1 h at room temperature. Protein expression was detected using an ECL kit (MA0186, Meilun, Dalian, China). The primary antibodies used for western blot analysis are listed as follows: β-tubulin (AC030, ABclonal, Wuhan, China), USP32 (sc-374465, Santa Cruz), DDX17 (sc-398168, Santa Cruz), GAPDH (AC054, ABclonal, Wuhan, China), Flag (AE121, ABclonal, Wuhan, China), Myc (AE070, ABclonal, Wuhan, China), HA(AE105, ABclonal, Wuhan, China) Phospho-p44/42 MAPK (Erk1/2) (Thr202/Tyr204)(#4370, CST, America), p44/42 MAPK (Erk1/2) (#9102, CST, America), Phospho-MEK1/2 (Ser221)(#2338, CST, America), MEK1/2(#8727, CST, America).

### Bioinformatics analysis

To elucidate the role of USP32 in the ubiquitination regulatory network, we integrated multi-source data for target screening. The Harper Lab Protein Interaction Database (curated by Wade Harper’s group at Harvard Medical School) was utilized as a high-confidence resource for protein-protein interactions and ubiquitination dynamics. This platform employs high-throughput mass spectrometry to systematically profile protein interactomes and post-translational modifications, providing critical insights into cancer and neurodegenerative diseases. From this database, we obtained co-immunoprecipitation mass spectrometry (co-IP-MS) data using USP32 as a bait. Additionally, we identified USP32-associated molecules significantly correlated in the TCGA hepatocellular carcinoma (HCC) cohort. Finally, potential substrates of USP32 were predicted using the Ubibrowser platform (http://ubibrowser.ncpsb.org), a computational tool for deubiquitinating enzyme-substrate interaction prediction. The intersection of these three datasets (Harper Lab co-IP-MS, TCGA-correlated genes, and Ubibrowser predictions) enabled the identification of high-confidence candidate interactors and substrates of USP32 in HCC.

### Co-immunoprecipitation

Cells were lysed in ice-cold RIPA buffer containing protease and phosphatase inhibitors. The lysates were cleared by centrifugation at 14,000 rpm for 15 min at 4°C. The supernatant was then incubated with the appropriate antibody overnight at 4°C with gentle rotation. Protein A/G agarose beads were added to the antibody-lysate mixture and incubated for an additional 2 h at 4°C. The beads were washed three times with RIPA buffer, and the immunoprecipitated proteins were eluted by boiling in SDS-PAGE loading buffer for 10 min. The eluted proteins were separated by SDS-PAGE and analyzed by Western blotting using specific antibodies.

### Immunofluorescence staining

Cells were first fixed with 4% paraformaldehyde for 15 min and permeabilized with 0.1% Triton X-100 in PBS for 10 min. After blocking with 5% BSA for 30 min, the cells were incubated with primary antibodies against the target proteins overnight at 4°C. The next day, the cells were washed with PBS and incubated with fluorescently labeled secondary antibodies for 1 h at room temperature. Nuclei were stained with DAPI for 5 min. Finally, the cells were mounted with mounting medium and visualized under a confocal fluorescence microscope.

### Immunohistochemistry

Tissue sections were dewaxed and rehydrated through a series of graded alcohols. Antigen retrieval was achieved by microwaving the slides in citrate buffer. Endogenous peroxidase activity was blocked with hydrogen peroxide, and non-specific binding was minimized by incubating the sections with serum-free protein block. Primary antibodies were applied overnight at 4°C, followed by incubation with species-specific secondary antibodies conjugated to horseradish peroxidase. Visualization was accomplished using a chromogenic substrate, and slides were counterstained with hematoxylin. Finally, sections were dehydrated, cleared, and mounted with permanent mounting medium.

### In vivo deubiquitination assay

For the in vivo deubiquitination assay, HA-Ub (WT/K48/K63), Myc-DDX17, Flag-USP32, or Flag-USP8^C743A^ plasmid was transfected into HEK293T cells for 48 h. Cells were then treated with 10 μM MG132 (133407-82-6, MCE) for 12 h. Then, cells were washed with pre-chilled phosphate-buffered saline (PBS) and lysed with RIPA extraction reagent. HA-ubiquitinated DDX17 was isolated using an anti-Myc antibody. The ubiquitination level of DDX17 was detected by Western blotting with an anti-HA antibody. In HCC cells, HA-Ub plasmid and USP32 siRNAs or plasmid were co-transfected. HA-ubiquitinated DDX17 was isolated using an anti-DDX17 antibody. The ubiquitination level of DDX17 was detected by Western blotting with an anti-HA antibody.

#### Combination index (CI) assay

HepG2 and HCCLM3 cells (WT or stably overexpressing USP32) were seeded in 96-well plates (5×10³ cells/well) and allowed to attach overnight. Cells were then treated with a concentration matrix of lenvatinib (ranging from 1 μM to 25 μM.) and selumetinib (0.2–4 μM) as single agents or in non-fixed-ratio combinations. After 24 h of incubation, cell viability was assessed using CCK-8 assay. The observed combination effect was calculated as the fractional inhibition of cell growth relative to vehicle control. CI values were calculated using CompuSyn software (version 1.0, ComboSyn, Inc., Paramus, NJ, USA), based on the Chou-Talalay method. CI < 1, CI = 1, and CI > 1 indicate synergistic, additive, and antagonistic effects, respectively. Fa-CI plot visually represent the synergistic effect of lenvatinib and selumetinib in USP32-overexpressing HCC cells.

### Statistical analyses

Statistical analyses were performed using SPSS (version 26.0; IBM Corp., Armonk, NY, USA) and GraphPad Prism (version 8.0; GraphPad Software, San Diego, CA, USA). Continuous data derived from at least three independent experiments are presented as mean ± standard deviation (SD). Differences between the two groups were assessed using a two-tailed, unpaired Student’s t-test. For comparisons among multiple groups, one-way or two-way ANOVA followed by Tukey’s comparisons test was performed. A *P*-value less than 0.05 was considered statistically significant.

## Results

### High USP32 expression predicts unfavorable clinicopathologic characteristics and prognosis of HCC patients

**Fig. 1:**
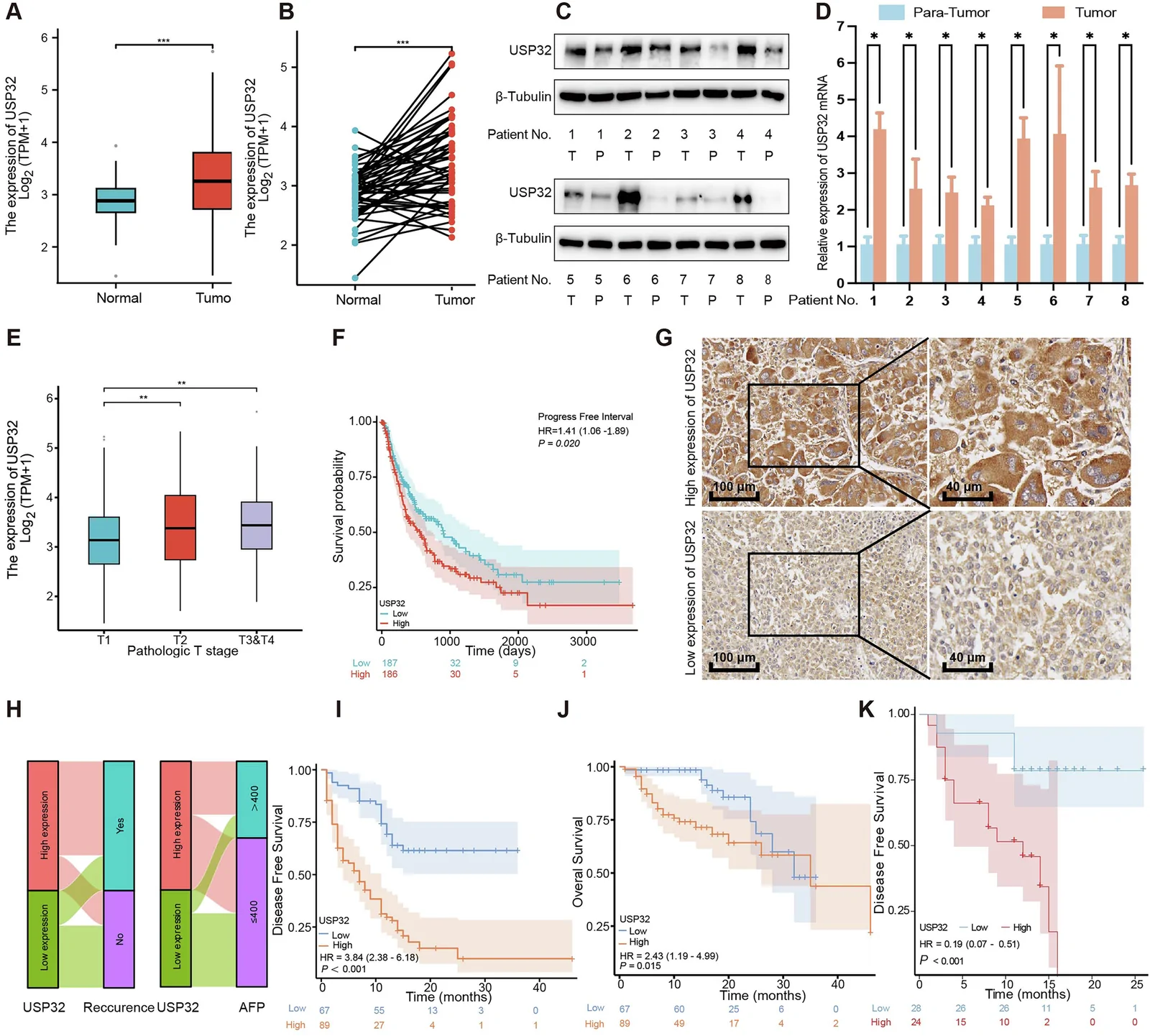
High USP32 expression predicts unfavorable clinicopathologic characteristics and prognosis of HCC patients. **A, B** USP32 expression showed higher in HCC tumor tissues compared to unpaired / matched normal tissues according to the TCGA database. **C, D** Protein and RNA lysates were extracted from 8 paired HCC tumors and adjacent non-tumor tissues obtained from our institutional cohort. Western blotting and qPCR analyses concordantly demonstrated upregulated USP32 expression in HCC (T:Tumor; P:Paracancerous). **E** USP32 correlated with a higher pathological T stage in the TCGA database. **F** HCC patients with higher expression of USP32 exhibited poor PFI according to the TCGA database. **G** 156 samples were used for IHC analysis. Representatively high and low expression of USP32 protein in HCC tissues were displayed. **H** Sankey diagrams demonstrated that high USP32 expression correlated with elevated serum AFP levels and a greater proportion of recurrence events. **I** Kaplan-Meier Survival analysis showed that HCC patients with high expression of USP32 had a poorer DFS. **J** Survival analysis showed HCC patients with high expression of USP32 had a poorer OS. **K** For patients with AFP < 20 μg/L, Kaplan-Meier analysis revealed significantly inferior disease-free survival (DFS) in the high USP32 expression subgroup (*P* < 0.001; HR = 0.19, 95% CI: 0.07–0.51).

Through analysis of The Cancer Genome Atlas (TCGA) database, we found that USP32 expression is significantly elevated in HCC tissues compared to normal liver tissues, irrespective of whether the samples were matched or unmatched (Figs. 1A, B). Consistently, RT-qPCR and western blotting demonstrated that USP32 mRNA and protein levels were upregulated in tumor tissues compared to matched adjacent non-tumor tissues collected at our institution (Figs. 1C and 1D). Notably, patients with high USP32 expression were significantly associated with more advanced clinical stages (Fig. 1E) and a shorter progression-free interval (PFI) in TCGA cohort (Fig. 1F).

Immunohistochemical (IHC) analysis was performed on tumor tissues obtained from 156 patients at our institution, revealing that 57% exhibited high USP32 expression (Fig. 1G). Analysis of baseline characteristics from our institutional cohort revealed that high USP32 expression correlated solely with elevated AFP levels (*P* = 0.021) and tumor recurrence (*P* < 0.001, Table 1). Sankey diagrams further visualized the association between high USP32 expression, elevated AFP, and increased recurrence probability (Fig. 1H). Survival analysis indicated that patients with elevated USP32 expression exhibited poorer DFS and OS outcomes (Figs. 1I, J). Cox regression analysis revealed that elevated USP32 expression independently predicted inferior DFS (Table 2). Notably, among patients with AFP < 20 μg/L, those with high USP32 expression demonstrated significantly worse disease-free survival (DFS) by Kaplan-Meier analysis (*P* < 0.001, Fig. 1K).

**Table 1:** Clinicopathologic characteristics of HCC patients according to the expression of USP32.

Variables	USP32		*P*-value
	High expression 89	low expression 67	
Gender, n (%)			0.308
male	67 (42.9%)	55 (35.3%)	
female	22 (14.1%)	12 (7.7%)	
Age, n (%)			0.739
≤50	27 (17.3%)	22 (14.1%)	
>50	62 (39.7%)	45 (28.8%)	
AFP, n (%)			0.021
≤400	52 (33.3%)	51 (32.7%)	
>400	37 (23.7%)	16 (10.3%)	
BCLC Grade, n (%)			0.676
0-A	61 (39.1%)	48 (30.8%)	
B	28 (17.9%)	19 (12.2%)	
Number, n (%)			0.967
Solitary	68 (43.6%)	51 (32.7%)	
Multiple	21 (13.5%)	16 (10.3%)	
Type of resection, n (%)			0.522
Anatomic	34 (21.8%)	29 (18.6%)	
Non-Anatomic	55 (35.3%)	38 (24.4%)	
Resection Margin, n (%)			0.647
≤1 cm	31 (19.9%)	21 (13.5%)	
>1 cm	58 (37.2%)	46 (29.5%)	
Diameter, mean ± sd	6.0921 ± 1.646	6.0567 ± 1.6093	0.893
Differentiation, n (%)			0.077
Ⅰ-Ⅱ	72 (46.2%)	61 (39.1%)	
Ⅲ-Ⅳ	17 (10.9%)	6 (3.8%)	
Capsule, n (%)			0.948
Complete	85 (54.5%)	65 (41.7%)	
Incomplete	4 (2.6%)	2 (1.3%)	
MVI, n (%)			0.113
Positive	39 (25%)	21 (13.5%)	
Negative	50 (32.1%)	46 (29.5%)	
Recurrence, n (%)			<0.001
Yes	65 (41.7%)	24 (15.4%)	
No	24 (15.4%)	43 (27.6%)

**Table 2:** Risk factors associated with DFS identified by Cox regression analysis.

Characteristics	Total(n)	HR(95% CI) Univariate analysis	*P*-Value	HR(95% CI) Multivariate analysis	*P*-Value
USP32	156				
high expression	89	Reference		Reference	
low expression	67	0.261 (0.162- 0.419)	< 0.001	0.154 (0.090 - 0.263)	< 0.001
Gender	156				
male	122	Reference			
female	34	1.248 (0.771 - 2.022)	0.368		
Age	156				
≤50	49	Reference			
>50	107	1.090 (0.693 - 1.714)	0.711		
AFP	156				
≤400	103	Reference		Reference	
>400	53	3.576 (2.325 - 5.502)	< 0.001	2.458 (1.541 - 3.922)	< 0.001
BCLC Grade	156				
0-A	109	Reference		Reference	
B	47	3.403 (2.161 - 5.358)	< 0.001	2.754 (1.575 - 4.814)	< 0.001
Number	156				
Solitary	119	Reference		Reference	
Multiple	37	2.704 (1.670 - 4.380)	< 0.001	1.189 (0.673 - 2.100)	0.552
Type of resection	156				
Anatomic	63	Reference		Reference	
Non-Anatomic	93	1.916 (1.241 - 2.958)	0.003	2.019 (1.183 - 3.446)	0.010
Resection Margin	156				
≤1 cm	52	Reference		Reference	
>1 cm	104	3.027 (1.816 - 5.047)	< 0.001	4.064 (2.320 - 7.118)	< 0.001
Diameter	156	1.090 (0.960 - 1.238)	0.183		
Differentiation	156				
I-II	133	Reference		Reference	
Ⅲ-IV	23	3.607 (2.176 - 5.981)	< 0.001	0.893 (0.482 - 1.654)	0.719
Capsule	156				
Complete	150	Reference		Reference	
Incomplete	6	9.145 (3.803 - 21.991)	< 0.001	4.071 (1.601 - 10.350)	0.003
MVI	156				
Positive	60	Reference		Reference	
Negative	96	0.214 (0.137 - 0.333)	< 0.001	0.354 (0.216 - 0.582)	< 0.001

### USP32 enhanced HCC cell proliferation, migration, and lenvatinib resistance

**Fig. 2:**
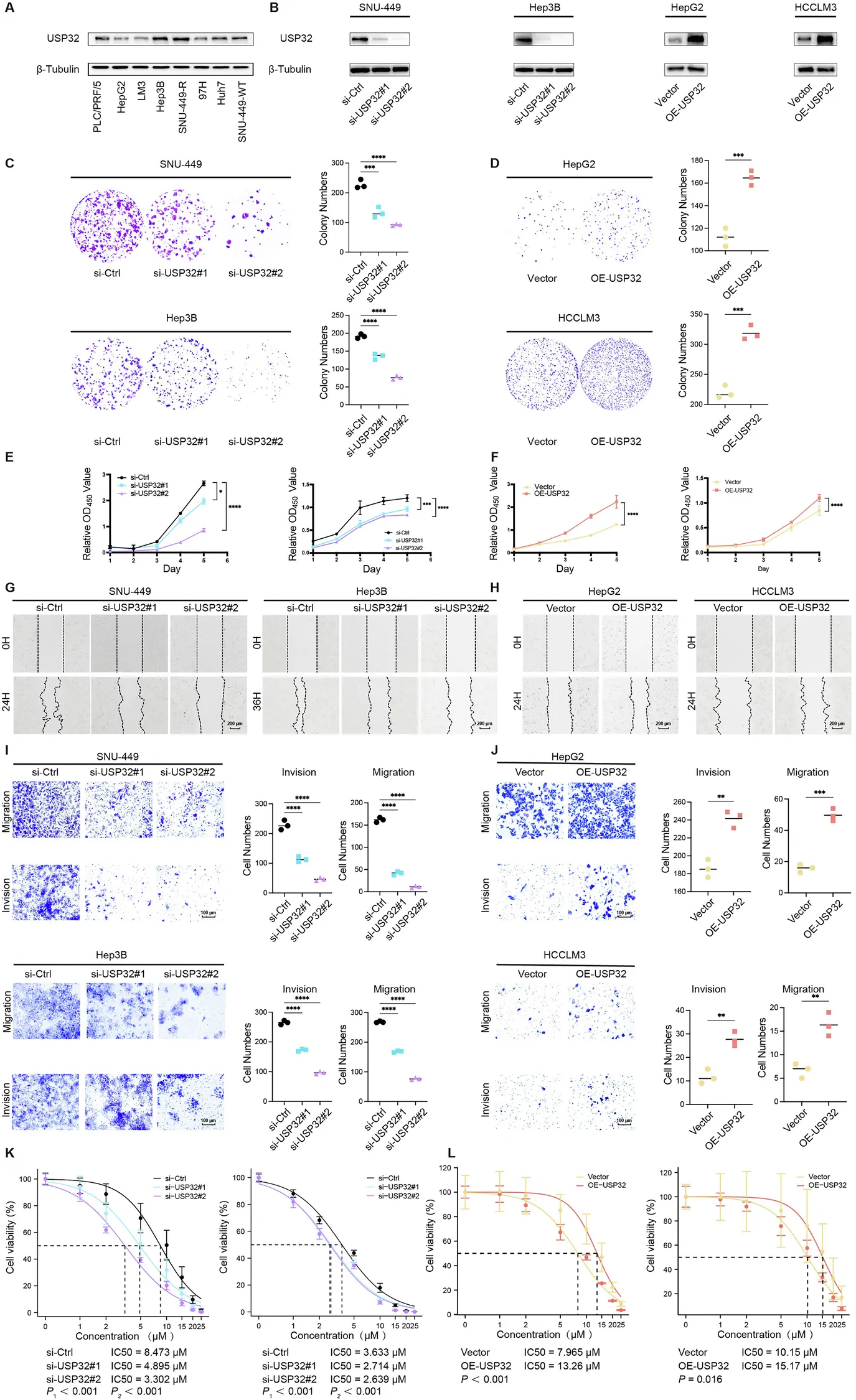
USP32 enhanced HCC cell proliferation, migration, and lenvatinib resistance. **A** USP32 expression was quantified in common HCC cell lines and lenvatinib-resistant SNU-449 cells.**B** Western blot analysis confirming USP32 knockdown in SNU-449 and Hep3B cells, and transient overexpression of USP32 in HepG2 and HCCLM3 cells. **C, D** Colony formation assays showing suppressed proliferation in USP32-knockdown SNU-449 and Hep3B cells and enhanced proliferation in USP32-overexpressing HepG2 and HCCLM3 cells. **E, F** CCK-8 assays indicating reduced proliferative capacity in USP32-knockdown SNU-449 and Hep3B cells and increased proliferation in USP32-overexpressing HepG2 and HCCLM3 cells. **G** Scratch wound healing assays demonstrating suppressed migration in USP32-knockdown SNU-449 and Hep3B cells. **H** Scratch wound healing assays showing enhanced migration in USP32-overexpressing HepG2 and HCCLM3 cells. **I** Transwell migration assays revealing impaired migratory capacity in USP32-knockdown SNU-449 and Hep3B cells. **J** Transwell migration assays indicating increased migration in USP32-overexpressing HepG2 and HCCLM3 cells. **K, L** CCK-8 assays following treatment with a concentration gradient of lenvatinib: USP32 knockdown enhanced drug sensitivity in SNU-449 and Hep3B cells (**I, J**; *P*_1_: si-Ctrl *vs.* si-USP32#1 and P_2_ si-Ctrl *vs.* si-USP32#2), while USP32 overexpression conferred increased resistance in HepG2 and HCCLM3 cells **K, L**. Statistical analysis, one-way ANOVA followed by Tukey’s comparisons test for **B** and **H**, two-tailed Student’s t test for **C** and I, two-way ANOVA followed by Tukey’s comparisons test for **D**, **E**, **J**, and **K**. * *P* < 0.05, ** *P* < 0.01, *** *P* < 0.001, **** *P* < 0.0001.

We first evaluated USP32 expression across a panel of HCC cell lines, including PLC/PRF/5, HepG2, HCCLM3, SNU-449, MHCC-97H, Huh-7, and Hep3B, as well as the lenvatinib-resistant SNU-449-R cell line. USP32 exhibited variable baseline expression among these cell lines, with SNU-449 and Hep3B identified as high-expressing, and HepG2 and HCCLM3 as low-expressing (Fig. 2A). Notably, USP32 expression was further elevated in SNU-449-R cells compared to its parental counterparts (Fig. 2A). To assess the biological functions of USP32 in HCC, we selected SNU-449 and Hep3B cells for knockdown using two non-overlapping siRNA, and HepG2 and HCCLM3 cells for transient overexpression via a USP32-expressing plasmid (Fig. 2B).

Knockdown of USP32 significantly inhibited the proliferative capacity of HCC cells, as demonstrated by colony formation and CCK-8 assays, whereas USP32 overexpression markedly enhanced HCC proliferation (Figs. 2C – F). Similarly, wound healing and transwell assays revealed that USP32 silencing substantially suppressed the migratory and invasive potential of HCC cells, while forced USP32 expression robustly promoted cell migration and invasion (Figs. 2G– J). Following treatment with a concentration gradient of lenvatinib, USP32 knockdown potentiated the lenvatinib sensitivity of HCC cells, whereas USP32 overexpression conferred resistance to lenvatinib (Figs. 2K and 2L).

To further confirm the functional relevance of USP32, we performed rescue experiments in HCC cell lines. In SNU-449 and Hep3B cells, which initially underwent USP32 knockdown, subsequent transient overexpression of USP32 partially reversed the suppressed proliferative, migratory, invasive, and lenvatinib sensitivity phenotypes (Figs. S1 and S2). Conversely, in HepG2 and HCCLM3 cells, which were first subjected to USP32 overexpression, co-transfection with USP32-targeting siRNAs attenuated the enhanced malignant behaviors (Figs. S1 and S2). These rescue assays further confirmed the essential role of USP32 in promoting HCC cell proliferation, migration, invasion, and resistance to lenvatinib.

### USP32 knockdown inhibits HCC progression and metastasis while potentiating lenvatinib efficacy in vivo

**Fig. 3:**
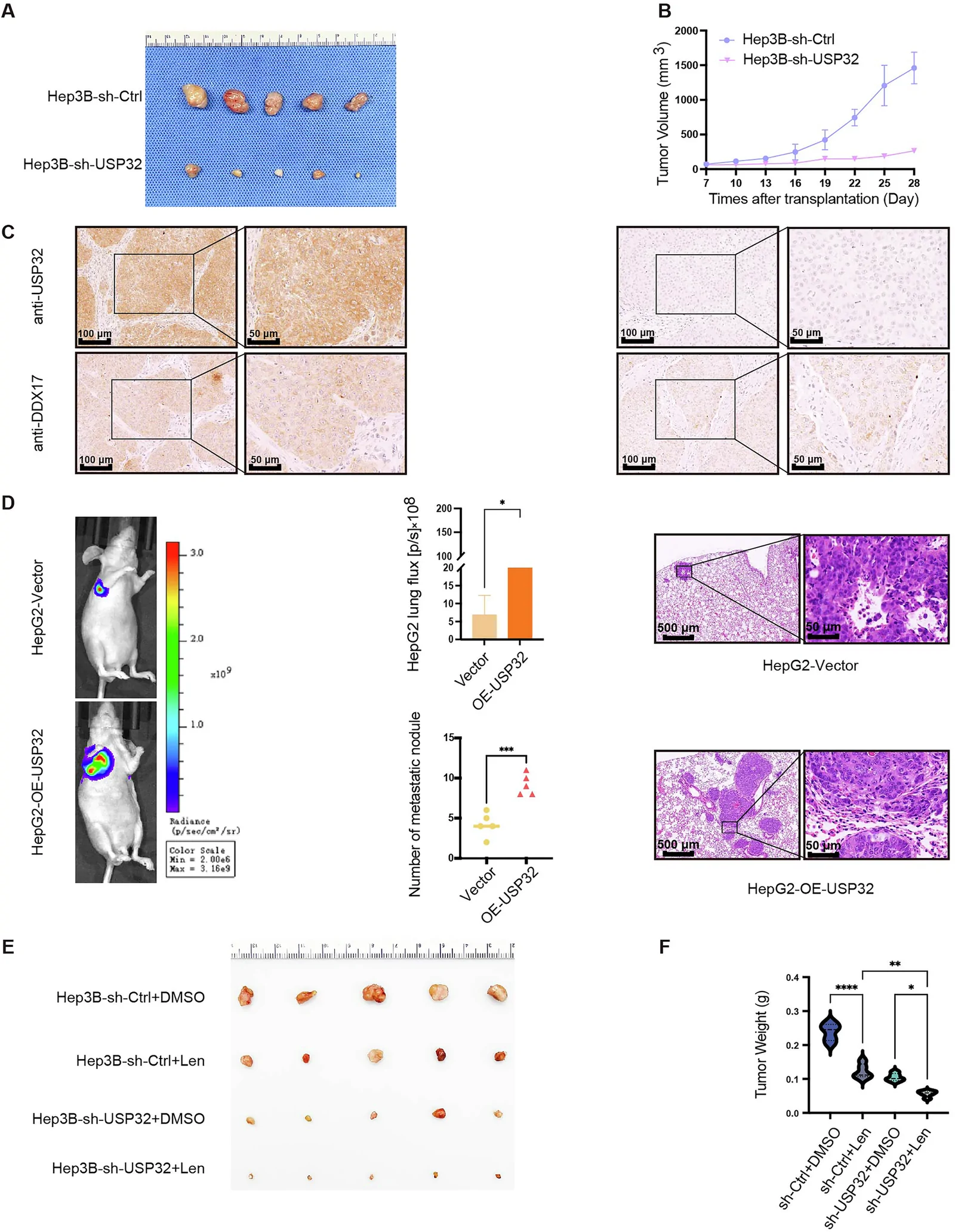
USP32 knockdown inhibits HCC progression and metastasis while potentiating lenvatinib efficacy in vivo. **A** Hep3B cells stably transfected with sh-Ctrl or sh-USP32 (5×10^6^ cells/mouse) were subcutaneously inoculated into 4-6-week-old nude mice (right flank). Tumor growth was monitored every 3 days for 4 weeks, followed by euthanasia and tumor harvest. Growth curves are shown in **B,** **C** HE staining and IHC for USP32 and DDX17 in subcutaneous tumors from both groups. **D** HepG2 cells stably expressing vector or USP32 (3×10^6^ cells/mouse) were intravenously injected into 4-6-week-old nude mice. Lung metastasis was assessed by bioluminescent imaging at 6 weeks. Lungs were harvested for HE staining. Quantification of lung metastatic nodules. **E** Subcutaneous tumor models were established. Following tumor formation, mice in the sh-Ctrl and sh-USP32 groups were treated via oral gavage with either lenvatinib or DMSO vehicle control. Mice were euthanized by cervical dislocation 4 weeks post-treatment initiation, and subcutaneous tumors were harvested. **F** Statistical analysis demonstrated that sh-USP32 mice treated with lenvatinib exhibited the most significant reduction in tumor weight. Statistical analysis, two-way ANOVA followed by Tukey’s comparisons test for **B**, two-tailed Student’s t test for **D**, one-way ANOVA followed by Tukey’s comparisons test for **F**. * *P* < 0.05, ** *P* < 0.01, *** *P* < 0.001, **** *P* < 0.0001.

Subcutaneous xenografts derived from USP32-knockdown Hep3B cells (sh-USP32) exhibited significantly reduced tumor volumes compared to control tumors (sh-Ctrl) (Figs. 3A, B). Immunohistochemistry (IHC) confirmed reduced USP32 and DDX17 protein levels in sh-USP32 tumors, consistent with in vitro results (Fig. 3C). In the pulmonary metastasis model via tail vein injection, USP32 overexpression markedly promoted lung metastasis, as evidenced by intensified bioluminescent signals and a greater number of metastatic foci confirmed by hematoxylin-eosin (HE) staining (Fig. 3D). Furthermore, mice bearing sh-USP32 or sh-Ctrl Hep3B xenografts were treated with lenvatinib (30 mg/kg/day) or vehicle (DMSO) via oral gavage. Lenvatinib elicited significantly greater tumor regression and lower tumor weight (Figs. 3E, F) in sh-USP32 tumors, indicating increased sensitivity to lenvatinib upon USP32 knockdown.

### USP32 stabilizes DDX17 through deubiquitylation mediated by the ubiquitin-proteasome pathway

**Fig. 4:**
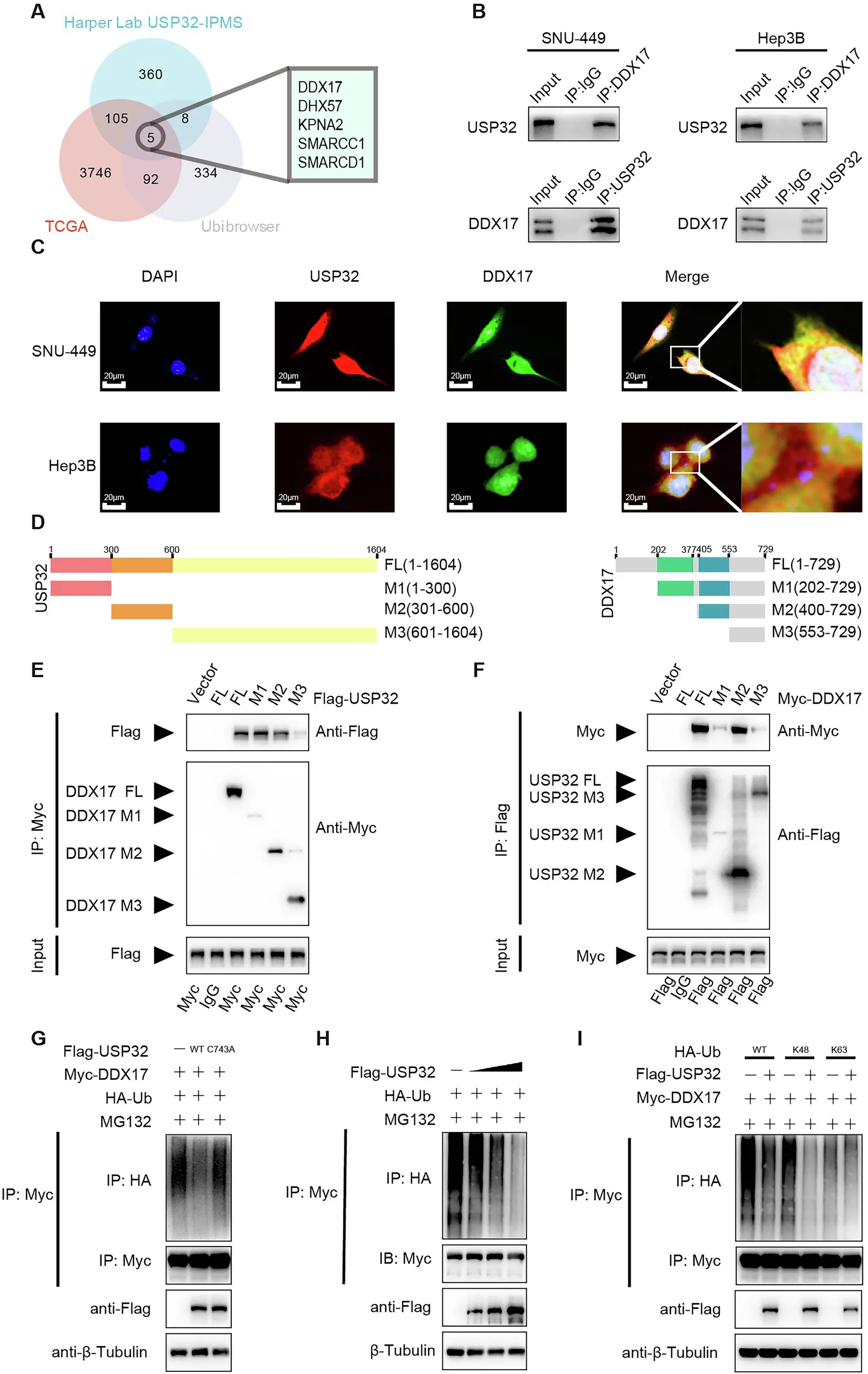
USP32 interacts with and deubiquitinates DDX17 in HCC cells. **A** Intersection analysis of USP32-associated proteins identified from three sources: co-immunoprecipitation mass spectrometry (co-IP-MS) data (Harper Lab Protein Interaction Database), TCGA-derived USP32-correlated genes, and predicted USP32 substrates from Ubibrowser. **B** Co-IP assay reveals association between endogenous USP32 and DDX17 in USP32-high-expressing SNU-449 and Hep3B cells. Cells were harvested with RIPA lysis buffer. **C** An immunofluorescence assay demonstrated partial colocalization of USP32 and DDX17 in SNU-449 and Hep3B cells. **D** Schematic representation of USP32 and DDX17 truncated mutants used in the study. **E** HEK293T cells were co-transfected with vectors expressing Myc-tagged full-length DDX17 or its truncation mutants and Flag-USP32. The cell lysates were immunoprecipitated with Myc antibody, followed by immunoblotting with the indicated antibodies. **F** HEK293T cells were co-transfected with vectors expressing Flag-tagged full-length USP32 or its truncation mutants and Myc-DDX17. Immunoprecipitation analysis of Flag and Myc in the indicated cells. **G** Immunoblot analysis of DDX17 ubiquitination in HEK-293T cells co-transfected with Myc-DDX17, HA-ubiquitin (Ub), and Flag-USP32 (wild-type or catalytically inactive mutant C743A). Ubiquitinated DDX17 was detected using anti-Myc antibody. **H** USP32 dose-dependently reduced DDX17 ubiquitination. HEK-293T cells expressing Myc-DDX17 and HA-Ub were transfected with increasing doses of Flag-USP32. **I** USP32 selectively cleaves K48-linked ubiquitin chains on DDX17. HEK-293T Cells co-expressing Myc-DDX17, Flag-USP32, and HA-tagged Ub mutants (WT, K48-only, or K63-only) were treated with 10 μM MG132 for 12 h. Ubiquitination levels were assessed by anti-HA immunoblotting after ubiquitin pull-down.

To investigate the underlying mechanism by which USP32 regulates malignant phenotypes in HCC, we integrated three datasets [1]. IP-MS data from the Harper Lab, a high-confidence resource for protein-protein interactions and ubiquitination dynamics; [2]. USP32-associated protein profiles from TCGA; and [3]. USP32 substrate predictions from Ubibrowser (http://ubibrowser.ncpsb.org/). Intersection analysis identified five candidate downstream substrates (Fig. 4A). Co-IP assays revealed that USP32 interacts with DDX17 in SNU-449 and Hep3B cells (Fig. 4B). Consistently, immunofluorescence staining was performed to demonstrate both USP32 and DDX17 mainly colocalized in the cytosol of HCC cells (Fig. 4C). To map the binding domains, we generated truncated mutants of USP32 and DDX17 based on previously structural studies (Fig. 4D) [21, 22]. Co-IP assays demonstrated that amino acids 300–600 of USP32 and 405-553 of DDX17 were respectively required and sufficient for their direct interaction under physiological conditions (Figs. 4E, F). These results indicate that USP32 and DDX17 formed a stable protein complex in HCC cells.

Given USP32’s function as a deubiquitinating enzyme (DUB), we next investigated whether DDX17 is a direct substrate of USP32. USP32 knockdown markedly increased the ubiquitination levels of DDX17. Conversely, overexpression of wild-type (WT) USP32, but not its catalytically inactive mutant (USP32^C743A^), significantly reduced DDX17 ubiquitination (Fig. 4G). In vivo ubiquitination assays further confirmed that USP32 reduced DDX17 ubiquitination in a dose-dependent manner (Fig. 4H). To determine the specific ubiquitin linkage types targeted by USP32, we performed deubiquitination assays using K48-only and K63-only ubiquitin mutants. The results revealed that USP32 selectively removed K48-linked polyubiquitin chains from DDX17, whereas K63-linked chains remained unaffected (Fig. 4I). Collectively, these findings identify USP32 as a DUB that removes K48-linked ubiquitin chains from DDX17.

**Fig. 5:**
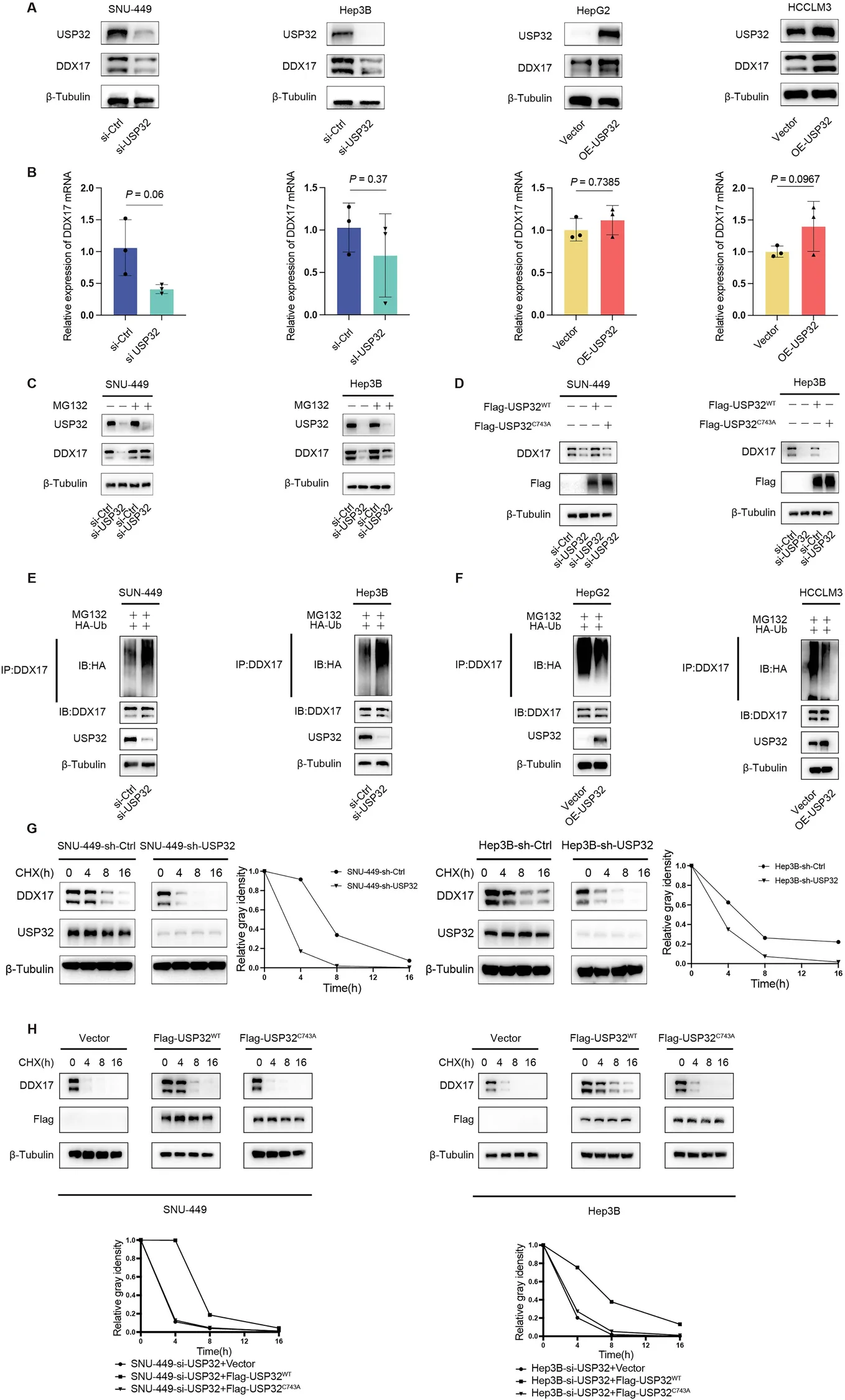
USP32 stabilizes DDX17 by preventing ubiquitin–proteasome-mediated degradation. **A** SNU-449 and Hep3B cells were transfected with siRNAUSP32, which reduced DDX17 protein levels, whereas transfection with USP32 overexpression plasmid in HepG2 and HCCLM3 cells increased DDX17 abundance. **B** q-PCR assays showed DDX17 mRNA levels remained unchanged across all cell lines under indicated manipulations. **C** SNU-449 and Hep3B cells transfected with the indicated siRNA were treated with or without the proteasome inhibitor MG132 (10 µM, 12 h), and then proteins were analyzed. **D** USP32 WT or C743A was introduced into SNU-449 and Hep3B cells together with USP32siRNA. DDX17 levels were measured. **E, F** Cells transfected with the indicated siRNA/overexpression plasmid and HA-Ub were treated with MG132 for 12 h before collection. DDX17 was immunoprecipitated with anti-DDX17 and immunoblotted with anti-HA. **G** SNU-449 and Hep3B cells transfected with USP32 siRNA were treated with cycloheximide (10 µg ml−1) and collected at the indicated times for western blot. Quantification of DDX17 levels relative to β-Tubulin is shown. **H** Half-life analysis of DDX17 in SNU-449 and Hep3B cells transfected with the indicated plasmids. Results shown are representative of 3 independent experiments.

Accordingly, we hypothesized that USP32 may regulate the turnover of DDX17 via the ubiquitination-mediated proteasomal degradation pathway. USP32 depletion decreased DDX17 levels, whereas overexpression increased DDX17 abundance (Fig. 5A), but no significant changes in DDX17 mRNA levels were observed under either condition (Fig. 5B), suggesting that USP32 regulates DDX17 at the post-transcriptional level. MG132 is a potent proteasome inhibitor that blocks ubiquitin-mediated protein degradation. In the presence of MG132, the reduction in DDX17 protein levels caused by USP32 knockdown was abolished, indicating that USP32 stabilizes DDX17 by preventing its proteasomal degradation (Fig. 5C). Consistently, the decrease of DDX17 induced by USP32 depletion was rescued by overexpression of USP32-WT but not USP32^C743A^ (Fig. 5D) [21].

Furthermore, in SNU-449 and Hep3B cells transfected with si-USP32 and HA-tagged ubiquitin (HA-Ub, to facilitate detection of ubiquitinated proteins), co-immunoprecipitation assays under MG132 treatment revealed that USP32 knockdown markedly increased DDX17 ubiquitination levels. Conversely, USP32 overexpression in HepG2 and HCCLM3 cells reduced DDX17 ubiquitination (Figs. 5E, F). To determine whether USP32 affects the stability of DDX17, we treated SNU-449 and Hep3B cells with cycloheximide (CHX), a protein synthesis inhibitor, and monitored DDX17 levels for 0, 4, 8, and 16 h. USP32 knockdown significantly shortened the half-life of DDX17 (Fig. 5G). In contrast, overexpression of USP32^WT^ but not USP32^C743A^ prolonged DDX17 stability (Fig. 5H). Collectively, these results indicate that USP32 stabilizes DDX17 by preventing its ubiquitin–proteasome-mediated degradation.

### USP32–DDX17 axis activates the MAPK pathway via the repression of miR-3163

Reciprocal rescue experiments further validated the USP32–DDX17 axis as a critical driver of HCC progression and lenvatinib resistance. In SNU-449 and Hep3B cells, USP32 overexpression-induced proliferation, migration, and lenvatinib resistance were abrogated by DDX17 knockdown. Conversely, in HepG2 and HCCLM3 cells, the tumor-suppressive effects of USP32 knockdown were rescued by DDX17 overexpression (Figs. S3 and S4). These results collectively underscore that USP32 exerts its oncogenic functions in a DDX17-dependent manner, functionally confirming the USP32–DDX17 regulatory axis in HCC.

**Fig. 6:**
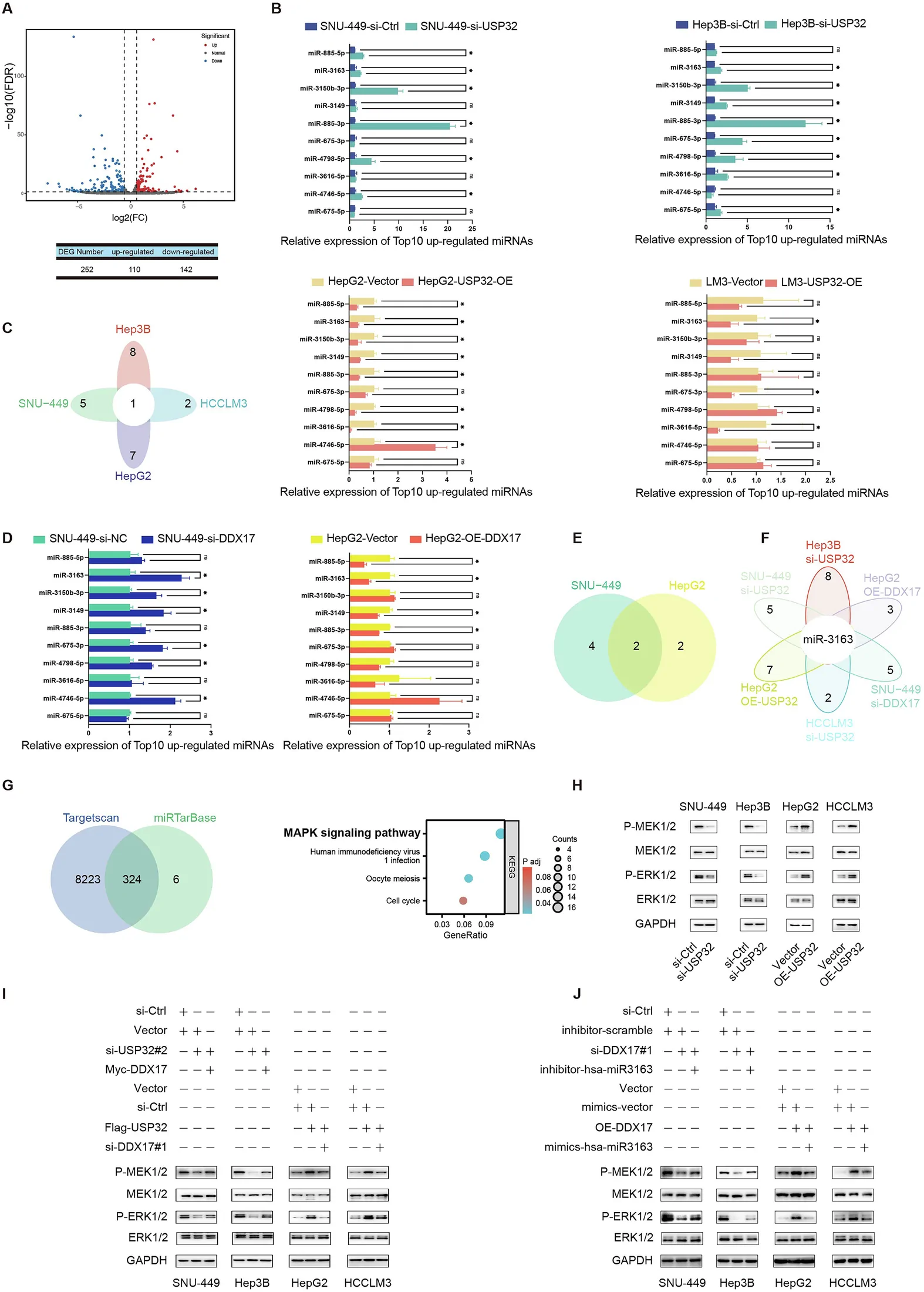
USP32–DDX17 axis activates the MAPK pathway via the repression of miR-3163. **A** Volcano plot of dysregulated miRNAs in response to USP32 knockdown. The twofold upregulated and downregulated miRNAs with adjusted *p* value < 0.05 are marked by red and blue, respectively. Statistical table of differentially expressed miRNAs. **B** RT‒qPCR determined the impacts of USP32 on the top 10 up-regulated miRNAs in indicated cells. **C** Venn diagram illustrating the intersection of significantly altered miRNAs across four HCC cell lines. **D** RT-qPCR analysis of candidate miRNA expression changes in SNU-449 cells following DDX17 knockdown and HepG2 cells following DDX17 overexpression. **E** Venn diagram showing overlapping miRNAs altered in both DDX17-knockdown **A** and DDX17-overexpressing **B** models. **F** Venn diagram identifying miR-3163 as the sole overlapping miRNA across four HCC cell lines with USP32 perturbations (knockdown/overexpression) and two cell lines with DDX17 manipulations. **G** Venn diagram of predicted miR-3163 targets from TargetScan and miRTarBase databases. KEGG pathway enrichment analysis of miR-3163 targets, highlighting significant association with the MAPK signaling pathway. **H** Immunoblot analysis of MAPK pathway components in USP32-knockdown SNU-449/Hep3B cells and USP32-overexpressing HepG2/HCCLM3 cells. **I** Immunoblot analysis of MAPK pathway alterations in SNU-449 and Hep3B cells with DDX17 knockdown ± miR-3163 inhibition, and in HepG2/HCCLM3 cells with DDX17 overexpression ± miR-3163 overexpression. **J** Immunoblot analysis of MAPK pathway alterations in SNU-449 and Hep3B cells with DDX17 knockdown ± miR-3163 inhibition, and in HepG2/HCCLM3 cells with DDX17 overexpression ± miR-3163 overexpression.

DDX17, a cofactor of the microprocessor complex, is known to participate in the recognition and cleavage of primary microRNA (miRNA) transcripts into precursor miRNA. In tumor contexts, dysregulation of the DDX17-associated microprocessor complex frequently suppresses tumor-suppressive miRNAs [18, 19]. To investigate how USP32-mediated stabilization of DDX17 contributes to miRNA dysregulation in HCC, we performed miRNA sequencing in USP32-deficient versus control Hep3B cells. Differential expression analysis revealed 110 upregulated and 142 downregulated miRNAs (Fold change ≥ 2, adjusted *p* < 0.05) (Fig. 6A). Among the top 10 upregulated miRNAs, RT-qPCR confirmed that USP32 knockdown significantly increased the expression of hsa-miR-3163 (hereafter referred to as miR-3163) in SNU-449 and Hep3B cells, while USP32 overexpression suppressed miR-3163 levels in HepG2 and HCCLM3 cells (*P* < 0.05) (Figs. 6B, C).

To determine whether DDX17 mediates USP32-driven regulation of miR-3163, we modulated DDX17 expression by knockdown in SNU-449 and overexpression in HepG2. (Fig. 6D). Intersectional analysis of miRNA profiles across these models identified miR-3163 as the most consistently regulated downstream candidate of the USP32–DDX17 axis (Figs. 6E, F). Previous studies have indicated that miR-3163 exerts tumor-suppressive effects in HCC [23, 24]. although its precise regulatory mechanism remains to be fully elucidated. To identify the downstream signaling pathways affected by the USP32–DDX17–miR-3163 axis, we used TargetScan and miRTarBase to predict 324 potential miR-3163 target genes, with KEGG enrichment analysis prioritizing the MAPK pathway (top-ranked, *P* < 0.05) (Fig. 6G). To functionally validate this finding, we assessed MAPK pathway activation under different USP32 and DDX17 expression conditions. USP32 knockdown in SNU-449 and Hep3B cells suppressed MAPK activity, as evidenced by reduced phosphorylation of ERK and MEK, whereas USP32 overexpression in HepG2 and HCCLM3 cells enhanced MAPK activation (Fig. 6H). Similarly, DDX17 depletion in SNU-449 and Hep3B cells attenuated MAPK signaling, which was restored by co-transfection with miR-3163 inhibitor (Fig. 6I). Conversely, DDX17 overexpression in HepG2 and HCCLM3 cells potentiated MAPK activation, which was abolished by co-expression of miR-3163 mimic (Fig. 6J). Collectively, these results demonstrate that the USP32–DDX17 axis activates the MAPK signaling pathway by repressing miR-3163.

### Dual inhibition of MAPK pathway in combination with lenvatinib synergistically overcomes drug resistance

**Fig. 7:**
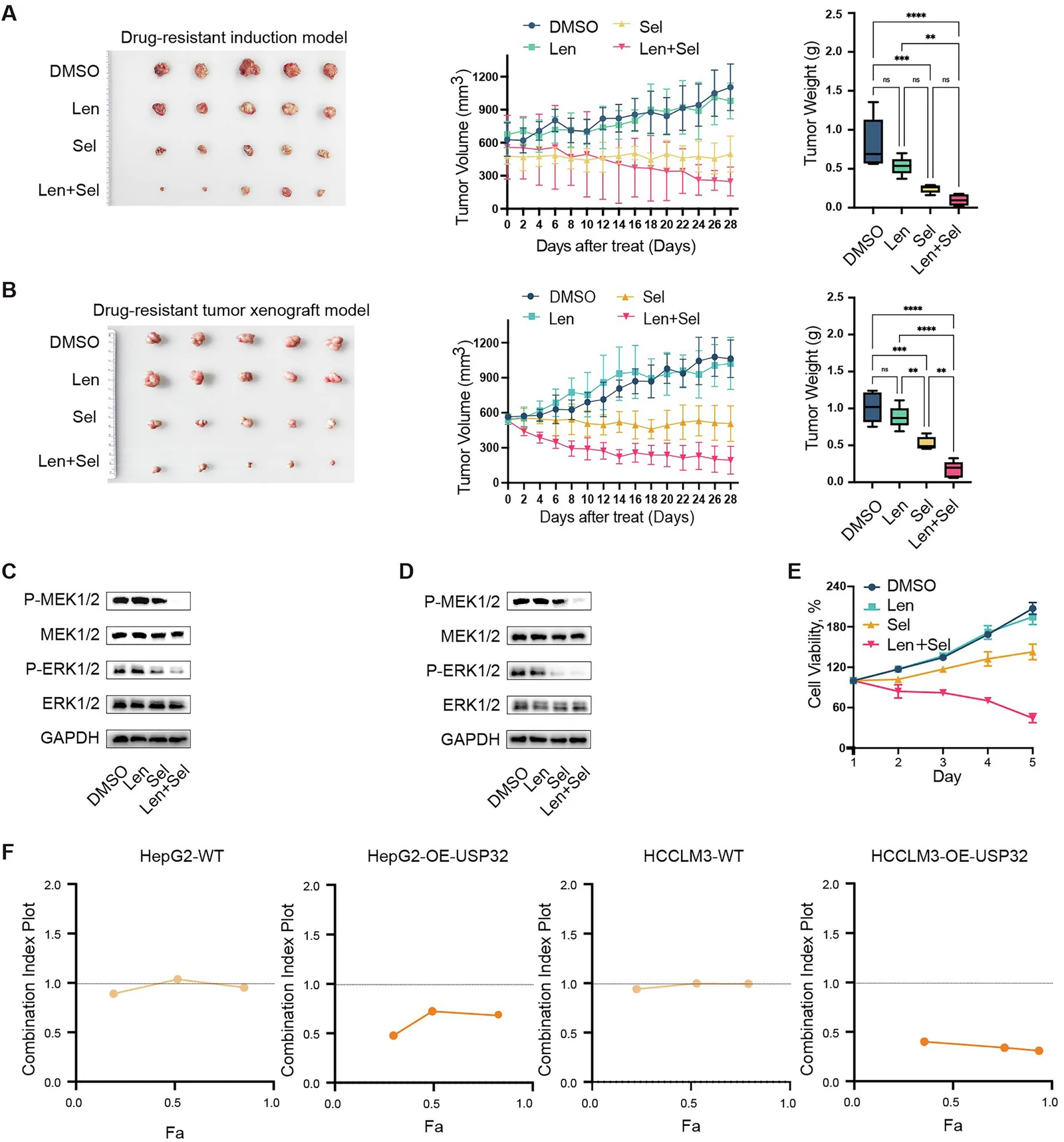
Dual inhibition of MAPK pathway in combination with lenvatinib synergistically overcomes drug resistance. **A** Induced lenvatinib resistance model: 4-6-week-old nude mice bearing HepG2-WT subcutaneous xenografts were treated with lenvatinib (30 mg/kg/day) via oral gavage for 4 weeks. Mice with progressive tumors (resistant cohort) were randomized into four groups receiving [1]. DMSO [2]. lenvatinib (30 mg/kg/day) [3]. selumetinib (MAPK inhibitor, 9.75 mg/kg/day), or [4]. lenvatinib + selumetinib. Tumor growth was monitored every 2 days until euthanasia at 4 weeks. **B** Transplanted resistance model: Lenvatinib-resistant tumors from **A** were excised and re-implanted subcutaneously into secondary mice, followed by identical four-group treatment. Tumor growth curves showing superior efficacy of lenvatinib-selumetinib combination versus monotherapy in both induced and transplanted resistance models. Lenvatinib and Selumetinib combination therapy significantly reduced tumor weights. **C, D** Immunoblot analysis of MAPK pathway activation in tumors from induced **A** and transplanted **B** resistance models, confirming pathway suppression in combination therapy groups. **E** CCK-8 assay assessing cell viability in lenvatinib-resistant SNU-449 cells. Cells were treated with DMSO (control), lenvatinib alone (10 μM), selumetinib alone (2 μM), or the combination of lenvatinib and selumetinib. Combination therapy significantly reversed lenvatinib resistance in hepatocellular carcinoma (HCC) cells. **F** Combination index (CI) analysis were performed in wild-type (WT) and USP32-overexpressing HCC cells (HepG2 and HCCLM3). Cells were treated with serial dilutions of lenvatinib, selumetinib, or their non-fixed-ratio combinations. Statistical analysis, one-way ANOVA followed by Tukey’s comparisons test for **A** and **B**. ns not significant, ** *P* < 0.01, *** *P* < 0.001, **** *P* < 0.0001.

Our previous findings demonstrated that MAPK pathway hyperactivation is a hallmark of lenvatinib-resistant hepatocellular carcinoma (HCC), consistent with established mechanisms of therapeutic resistance [25–27]. To investigate whether MAPK inhibition could restore lenvatinib sensitivity, we established two in vivo resistance models [1]. an acquired resistance model generated by prolonged lenvatinib treatment of HepG2-WT xenografts, and [2]. a transplant model engrafted with resistant tumors from the former. Both models were randomized into four treatment arms: DMSO (vehicle), lenvatinib (30 mg/kg/day), selumetinib (MAPK inhibitor, 9.75 mg/kg/day), or a combination of lenvatinib and selumetinib. Despite mild treatment-related toxicity such as body weight loss (Figs. S5A–S5D), the combination therapy significantly reduced tumor volume and weight in both resistance models, outperforming either monotherapy and effectively overcoming lenvatinib resistance (Fig. 7A, B, Figs. S5E and S5F). Moreover, western blot analysis of resected tumors further validated the potent suppression of MAPK pathway in the combination therapy group, as evidenced by reduced levels of phosphorylated ERK1/2 and MEK1/2 (Fig. 7C, D). Validation of combination therapy in SNU-449-R cells was approved by CCK-8 assay, and the synergistic effect of lenvatinib and selumetinib in reversing acquired resistance was recapitulated in this resistant cell line (Fig. 7E). Additionally, combination index (CI) analysis was conducted in HepG2 and HCCLM3 cells. In WT HepG2 and HCCLM3 cells, the CI values ranged from 0.89 to 1.04 for HepG2 and 0.94 to 1.00 for HCCLM3 across all tested concentrations, indicating no clear synergy (predominantly additive effects); in sharp contrast, USP32-overexpressing cells exhibited robust synergy, with CI values not exceeding 0.72 for HepG2 and 0.40 for HCCLM3 at multiple concentration combinations (peak CI = 0.72 at 5 μM lenvatinib + 0.8 μM selumetinib for HepG2 and 0.40 at 2 μM lenvatinib + 0.4 μM selumetinib for HCCLM3). CI analysis confirmed that lenvatinib plus selumetinib synergized specifically in USP32-overexpressing cells (Fig. 7F), thereby providing mechanistic support for the enhanced anti-tumor efficacy.

## Discussion

In this study, we identify USP32 as a previously unrecognized oncogenic drive in HCC that promotes tumor progression and lenvatinib resistance through deubiquitination-mediated stabilization of the RNA helicase DDX17. Stabilized DDX17 represses the tumor-suppressive microRNA miR-3163, resulting in sustained activation of the MAPK signaling pathway. By integrating patient-derived transcriptomic data, in vitro and in vivo functional assays, and mechanistic interrogation, we delineate a USP32/DDX17/miR-3163/MAPK regulatory axis that contributes to HCC malignancy and lenvatinib resistance. These findings provide mechanistic insight into the modulation of the post-translational modification via ubiquitin-proteasome pathway and identify the MAPK pathway as an actionable target for combinational therapy to overcome lenvatinib resistance in HCC.

Accumulating evidence has emphasized the pivotal role of the ubiquitin-proteasome system in regulating protein stability and oncogenic signaling in cancer. DUBs such as USP7, which stabilizes KDM5B, or USP9X, which regulates p53, have been implicated in other malignancies, highlighting the oncogenic potential of selective substrate deubiquitination [28, 29]. However, the contribution of deubiquitinating enzymes (DUBs) to post-translational regulation in HCC remains underexplored. In our study, the observed overexpression of USP32 in HCC and its association with poor clinical outcomes support its potential role as a prognostic biomarker and therapeutic target. Our study expands this understanding by identifying USP32 as a critical upstream regulator that selectively stabilizes DDX17 through removal of K48-linked polyubiquitin chains, thereby prolonging its half-life (Fig. 5). The USP32–DDX17 interaction constitutes a distinct regulatory node in HCC pathogenesis.

DDX17, an RNA helicase critical for pri-miRNA processing, has previously been implicated in promoting tumor progression and therapeutic resistance in multiple cancer types, including HCC. While DDX17 has been linked to alternative pathways such as Wnt/β-catenin signaling in other cancer contexts [30]. our findings highlight the mechanistic versatility of RNA helicases in regulating context-dependent transcriptional and post-transcriptional networks. We demonstrate that USP32-mediated stabilization of DDX17 suppresses the tumor-suppressive microRNA miR-3163, leading to sustained activation of the MAPK signaling cascade. Importantly, the link between miR-3163 suppression and MAPK pathway activation provides a direct molecular explanation for how USP32 contributes to lenvatinib resistance. Given the well-established role of MAPK hyperactivation in lenvatinib resistance [31–34]. This axis may represent a key adaptive mechanism in HCC. These results support a model in which aberrant deubiquitination not only alters protein turnover but also rewires transcriptional and post-transcriptional networks that converge on clinically actionable pathways.

Our preclinical models recapitulated the phenotype of lenvatinib resistance, demonstrating that the MAPK inhibitor selumetinib restores lenvatinib sensitivity even in USP32-high tumors (Fig. 7). The previous findings indicate that elevated USP32 expression correlates with aggressive tumor phenotypes and poor prognosis, suggesting its potential utility as a biomarker for identifying patients at higher risk of lenvatinib resistance. In this context, immunohistochemical or transcriptomic assessment of USP32 expression could serve as a feasible strategy for patient stratification in clinical settings. Although USP32 itself may be challenging to target pharmacologically due to structural constraints and potential off-target effects, its downstream consequence of MAPK pathway activation presents a more tractable and clinically actionable vulnerability. Patients with high USP32 expression may particularly benefit from early incorporation of MAPK inhibitors in combination with lenvatinib, aiming to prevent or delay the emergence of drug resistance. Future investigations incorporating clinical HCC specimens are needed to validate USP32 as a predictive biomarker for lenvatinib resistance and to assess its utility in guiding combination strategies involving MAPK inhibitors.

In summary, as shown in graphic abstract (Fig. S6), this study identifies the USP32/DDX17/miR-3163/MAPK signaling axis that promotes tumor progression and lenvatinib resistance via post-translational stabilization of oncogenic pathways. These findings provide mechanistic insight into ubiquitin-dependent lenvatinib resistance and demonstrate that the MEK inhibitor selumetinib synergistically suppresses resistant tumor growth in vivo, offering an actionable strategy to overcome lenvatinib resistance.

## Supplementary information

Supplementary Material

Original Data

## Data Availability

All data and materials used in this study are available from the corresponding authors upon reasonable request.

## References

[1] Toh MR, Wong EYT, Wong SH, Ng AWT, Loo LH, Chow PK, et al. Global Epidemiology and Genetics of Hepatocellular Carcinoma. Gastroenterology. 2023;164:766–82. 36738977 10.1053/j.gastro.2023.01.033

[2] Grandhi MS, Kim AK, Ronnekleiv-Kelly SM, Kamel IR, Ghasebeh MA, Pawlik TM. Hepatocellular carcinoma: From diagnosis to treatment. Surg Oncol. 2016;25:74–85. 27312032 10.1016/j.suronc.2016.03.002

[3] Liu L, Dai H, Wu Y, Li B, Yi J, Xu C, et al. In vitro and in vivo mechanism of hepatocellular carcinoma inhibition by β-TCP nanoparticles. Int J Nanomed. 2019;14:3491–502. 10.2147/IJN.S193192 PMC6526184 31190806

[4] Wei L, Lee D, Law CT, Zhang MS, Shen J, Chin DW, et al. Genome-wide CRISPR/Cas9 library screening identified PHGDH as a critical driver for Sorafenib resistance in HCC. Nat Commun. 2019;10:4681. 31615983 10.1038/s41467-019-12606-7 PMC6794322

[5] Kudo M, Finn RS, Qin S, Han KH, Ikeda K, Piscaglia F, et al. Lenvatinib versus sorafenib in first-line treatment of patients with unresectable hepatocellular carcinoma: a randomised phase 3 non-inferiority trial. Lancet. 2018;391:1163–73. 29433850 10.1016/S0140-6736(18)30207-1

[6] Matsui J, Yamamoto Y, Funahashi Y, Tsuruoka A, Watanabe T, Wakabayashi T, et al. E7080, a novel inhibitor that targets multiple kinases, has potent antitumor activities against stem cell factor producing human small cell lung cancer H146, based on angiogenesis inhibition. Int J Cancer. 2008;122:664–71. 17943726 10.1002/ijc.23131

[7] Matsui J, Funahashi Y, Uenaka T, Watanabe T, Tsuruoka A, Asada M. Multi-kinase inhibitor E7080 suppresses lymph node and lung metastases of human mammary breast tumor MDA-MB-231 via inhibition of vascular endothelial growth factor-receptor (VEGF-R) 2 and VEGF-R3 kinase. Clin Cancer Res. 2008;14:5459–65. 18765537 10.1158/1078-0432.CCR-07-5270

[8] Pan J, Zhang M, Dong L, Ji S, Zhang J, Zhang S, et al. Genome-Scale CRISPR screen identifies LAPTM5 driving lenvatinib resistance in hepatocellular carcinoma. Autophagy. 2023;19:1184–98. 36037300 10.1080/15548627.2022.2117893 PMC10012959

[9] Takahashi M, Araki T, Yashima H, Nagamine A, Nagano D, Yamamoto K. Increased c‑SRC expression is involved in acquired resistance to lenvatinib in hepatocellular carcinoma. Oncol Lett. 2023;26:529. 38020292 10.3892/ol.2023.14116 PMC10654551

[10] Zou T, Lin Z. The Involvement of Ubiquitination Machinery in Cell Cycle Regulation and Cancer Progression. Int J Mol Sci. 2021;22:5754. 34072267 10.3390/ijms22115754 PMC8198665

[11] Dang F, Nie L, Wei W. Ubiquitin signaling in cell cycle control and tumorigenesis. Cell Death Differ. 2021;28:427–38. 33130827 10.1038/s41418-020-00648-0 PMC7862229

[12] Yau RG, Doerner K, Castellanos ER, Haakonsen DL, Werner A, Wang N, et al. Assembly and Function of Heterotypic Ubiquitin Chains in Cell-Cycle and Protein Quality Control. Cell. 2017;171:918–.e920. 29033132 10.1016/j.cell.2017.09.040 PMC5669814

[13] Lee JC, Peter ME. Regulation of apoptosis by ubiquitination. Immunol Rev. 2003;193:39–47. 12752669 10.1034/j.1600-065x.2003.00043.x

[14] Gao L, Zhang W, Shi XH, Chang X, Han Y, Liu C, et al. The mechanism of linear ubiquitination in regulating cell death and correlative diseases. Cell Death Dis. 2023;14:659. 37813853 10.1038/s41419-023-06183-3 PMC10562472

[15] Sampson C, Wang Q, Otkur W, Zhao H, Lu Y, Liu X, et al. The roles of E3 ubiquitin ligases in cancer progression and targeted therapy. Clin Transl Med. 2023;13:e1204. 36881608 10.1002/ctm2.1204 PMC9991012

[16] Li S, Yang L, Ding X, Sun H, Dong X, Yang F, et al. USP32 facilitates non-small cell lung cancer progression via deubiquitinating BAG3 and activating RAF-MEK-ERK signaling pathway. Oncogenesis. 2024;13:27. 39030175 10.1038/s41389-024-00528-z PMC11271578

[17] Dou N, Hu Q, Li L, Wu Q, Li Y, Gao Y. USP32 promotes tumorigenesis and chemoresistance in gastric carcinoma via upregulation of SMAD2. Int J Biol Sci. 2020;16:1648–57. 32226309 10.7150/ijbs.43117 PMC7097920

[18] Wu KJ. The role of miRNA biogenesis and DDX17 in tumorigenesis and cancer stemness. Biomed J. 2020;43:107–14. 32513392 10.1016/j.bj.2020.03.001 PMC7283569

[19] Zhao G, Wang Q, Zhang Y, Gu R, Liu M, Li Q. DDX17 induces epithelial-mesenchymal transition and metastasis through the miR-149-3p/CYBRD1 pathway in colorectal cancer. Cell Death Dis. 2023;14:1. 36593242 10.1038/s41419-022-05508-y PMC9807641

[20] Zhou HZ, Li F, Cheng ST, Xu Y, Deng HJ, Gu DY, et al. DDX17-regulated alternative splicing that produced an oncogenic isoform of PXN-AS1 to promote HCC metastasis. Hepatology. 2022;75:847–65. 34626132 10.1002/hep.32195 PMC9304246

[21] Li C, Gao Z, Cui Z, Liu Z, Bian Y, Sun H, et al. Deubiquitylation of Rab35 by USP32 promotes the transmission of imatinib resistance by enhancing exosome secretion in gastrointestinal stromal tumours. Oncogene. 2023;42:894–910. 36725886 10.1038/s41388-023-02600-1

[22] Jin C, Han-Hua D, Qiu-Meng L, Deng N, Peng-Chen D, Jie M, et al. MTDH-stabilized DDX17 promotes tumor initiation and progression through interacting with YB1 to induce EGFR transcription in Hepatocellular Carcinoma. Oncogene. 2023;42:169–83. 36385375 10.1038/s41388-022-02545-x

[23] Yang B, Wang C, Xie H, Wang Y, Huang J, Rong Y, et al. MicroRNA-3163 targets ADAM-17 and enhances the sensitivity of hepatocellular carcinoma cells to molecular targeted agents. Cell Death Dis. 2019;10:784. 31611551 10.1038/s41419-019-2023-1 PMC6791891

[24] Shi C, Yang Q, Pan S, Lin X, Xu G, Luo Y, et al. LncRNA OIP5-AS1 promotes cell proliferation and migration and induces angiogenesis via regulating miR-3163/VEGFA in hepatocellular carcinoma. Cancer Biol Ther. 2020;21:604–14. 32329664 10.1080/15384047.2020.1738908 PMC7515459

[25] Delire B, Stärkel P. The Ras/MAPK pathway and hepatocarcinoma: pathogenesis and therapeutic implications. Eur J Clin Invest. 2015;45:609–23. 25832714 10.1111/eci.12441

[26] Guo J, Zhao J, Xu Q, Huang D. Resistance of Lenvatinib in Hepatocellular Carcinoma. Curr Cancer Drug Targets. 2022;22:865–78. 36267045 10.2174/1568009622666220428111327

[27] Claesson-Welsh L, Welsh M. VEGFA and tumour angiogenesis. J Intern Med. 2013;273:114–27. 23216836 10.1111/joim.12019

[28] Zhang B, Li J, Wang Y, Liu X, Yang X, Liao Z, et al. Deubiquitinase USP7 stabilizes KDM5B and promotes tumor progression and cisplatin resistance in nasopharyngeal carcinoma through the ZBTB16/TOP2A axis. Cell Death Differ. 2024;31:309–21. 38287116 10.1038/s41418-024-01257-x PMC10923876

[29] Chen E, Li E, Liu H, Zhou Y, Wen L, Wang J, et al. miR-26b enhances the sensitivity of hepatocellular carcinoma to Doxorubicin via USP9X-dependent degradation of p53 and regulation of autophagy. Int J Biol Sci. 2021;17:781–95. 33767588 10.7150/ijbs.52517 PMC7975695

[30] Liu GB, Cheng YX, Li HM, Liu Y, Sun LX, Wu Q, et al. Ghrelin promotes cardiomyocyte differentiation of adipose tissue‑derived mesenchymal stem cells by DDX17‑mediated regulation of the SFRP4/Wnt/β‑catenin axis. Mol Med Rep. 2023;28:164. 37449526 10.3892/mmr.2023.13050 PMC10407612

[31] Song Y, Bi Z, Liu Y, Qin F, Wei Y, Wei X. Targeting RAS-RAF-MEK-ERK signaling pathway in human cancer: Current status in clinical trials. Genes Dis. 2023;10:76–88. 37013062 10.1016/j.gendis.2022.05.006 PMC10066287

[32] Ullah R, Yin Q, Snell AH, Wan L. RAF-MEK-ERK pathway in cancer evolution and treatment. Semin Cancer Biol. 2022;85:123–54. 33992782 10.1016/j.semcancer.2021.05.010

[33] Yuan J, Dong X, Yap J, Hu J. The MAPK and AMPK signalings: interplay and implication in targeted cancer therapy. J Hematol Oncol. 2020;13:113. 32807225 10.1186/s13045-020-00949-4 PMC7433213

[34] Cargnello M, Roux PP. Activation and function of the MAPKs and their substrates, the MAPK-activated protein kinases. Microbiol Mol Biol Rev. 2011;75:50–83. 21372320 10.1128/MMBR.00031-10 PMC3063353

