# Supplementary material for: Deubiquitinase USP32 stabilizes DDX17 to promote tumor progression and lenvatinib resistance in hepatocellular carcinoma via the miR-3163/MAPK pathway
Source: Cell Death Dis. 2026 Jun 25;17(1):789. doi: 10.1038/s41419-026-09040-1 (PMC13558724; doi:10.1038/s41419-026-09040-1)

Figure 1C

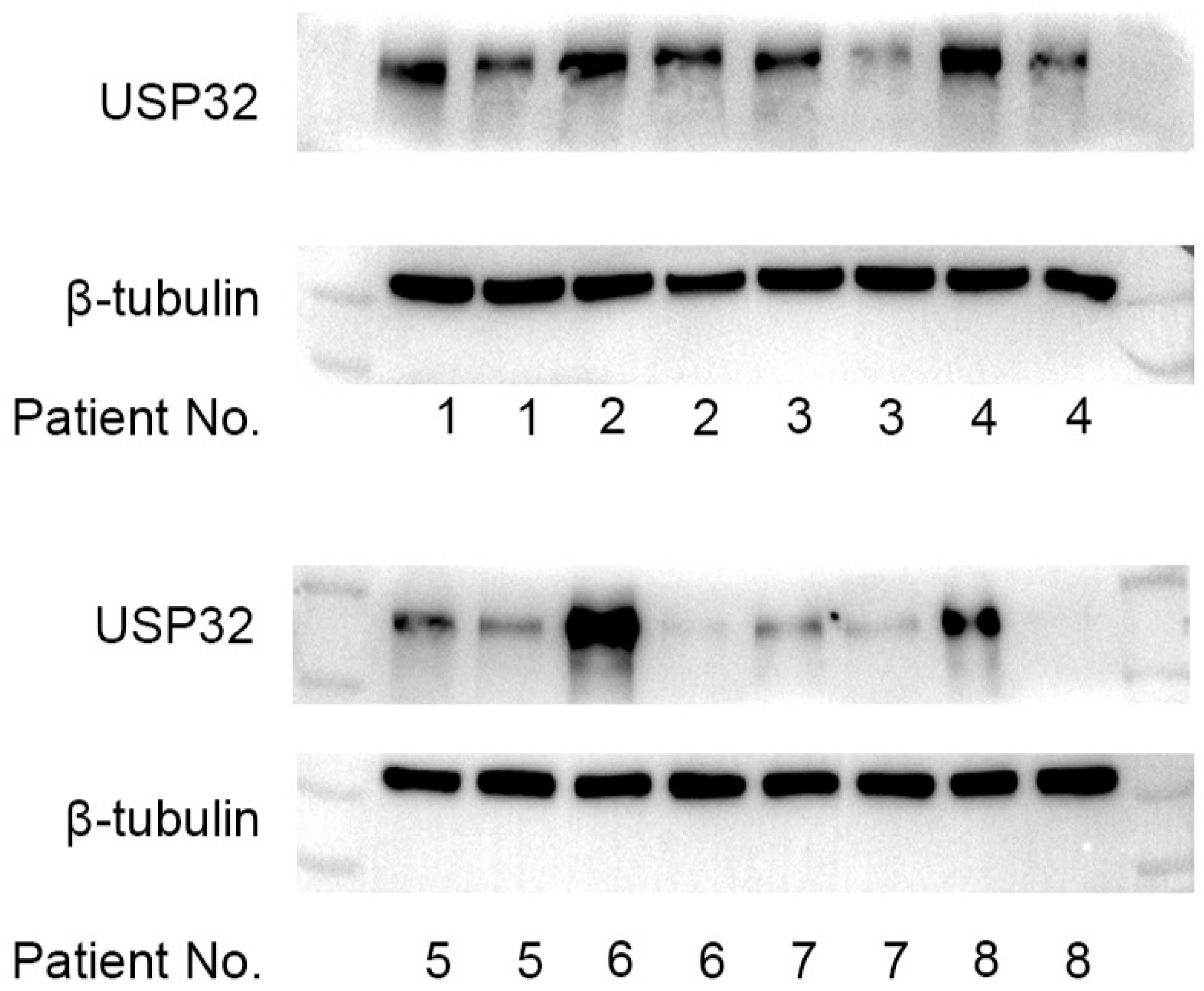

Figure 2A

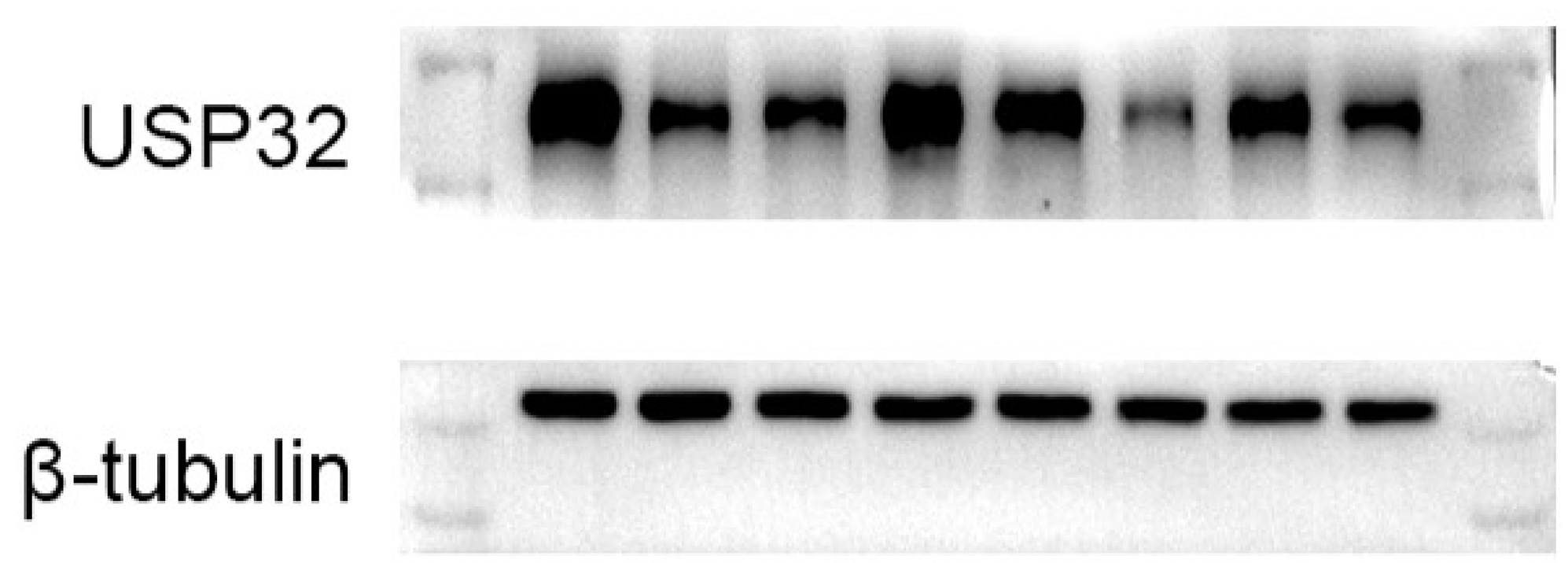

Figure 2B

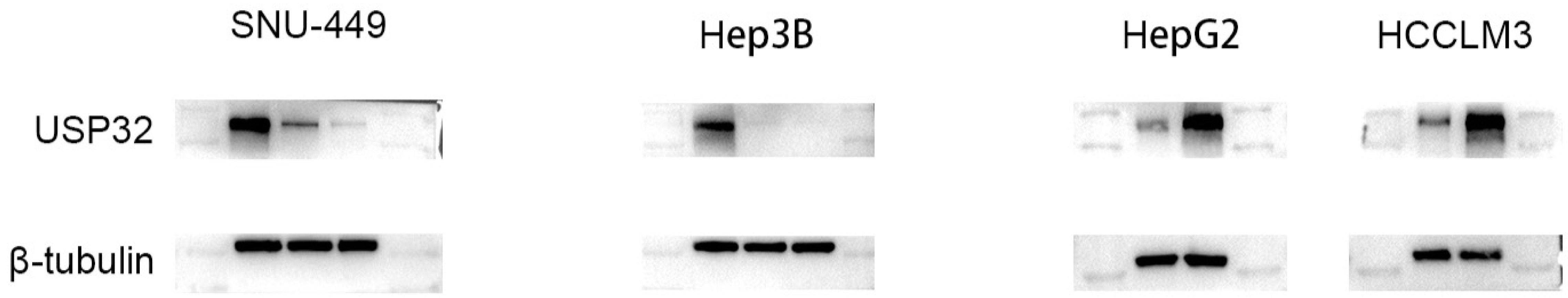

Figure 3B

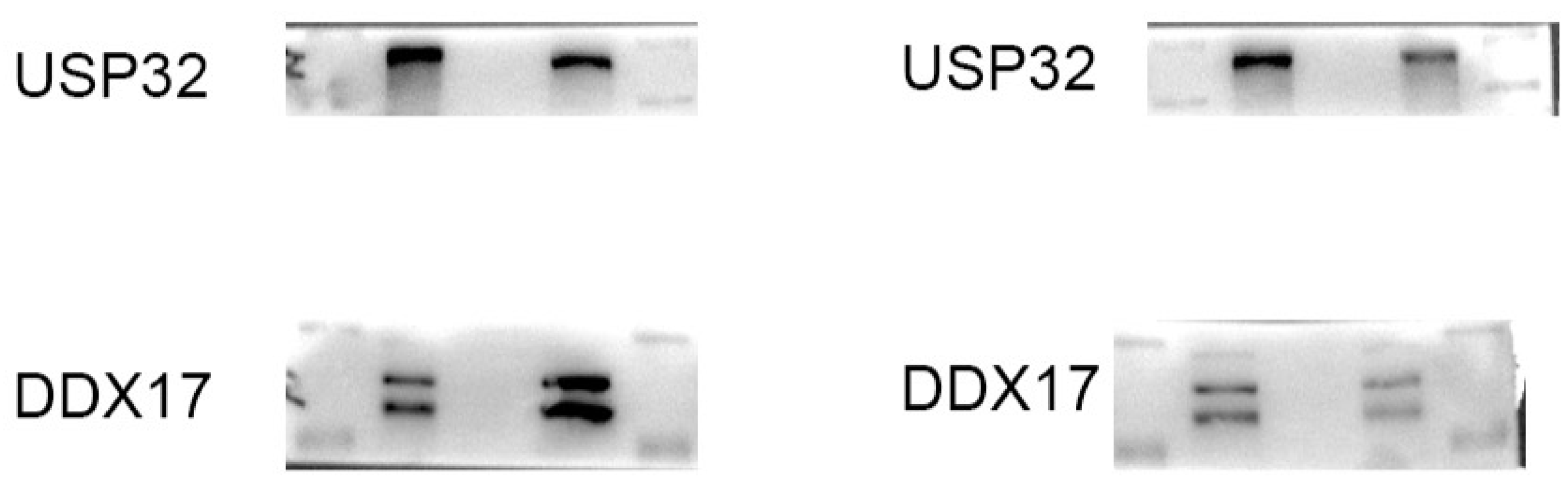

Figure 3E

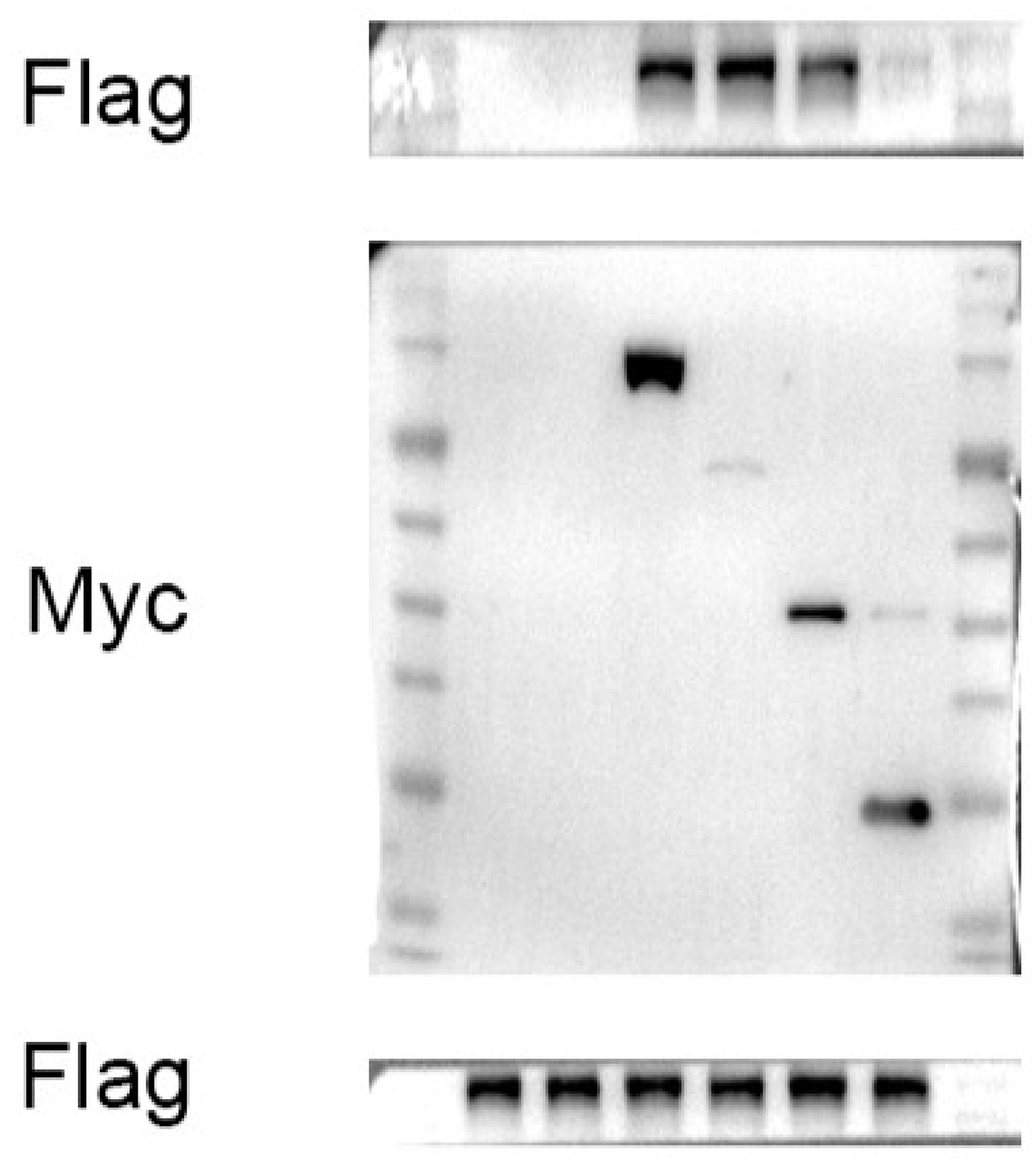

Figure 3F

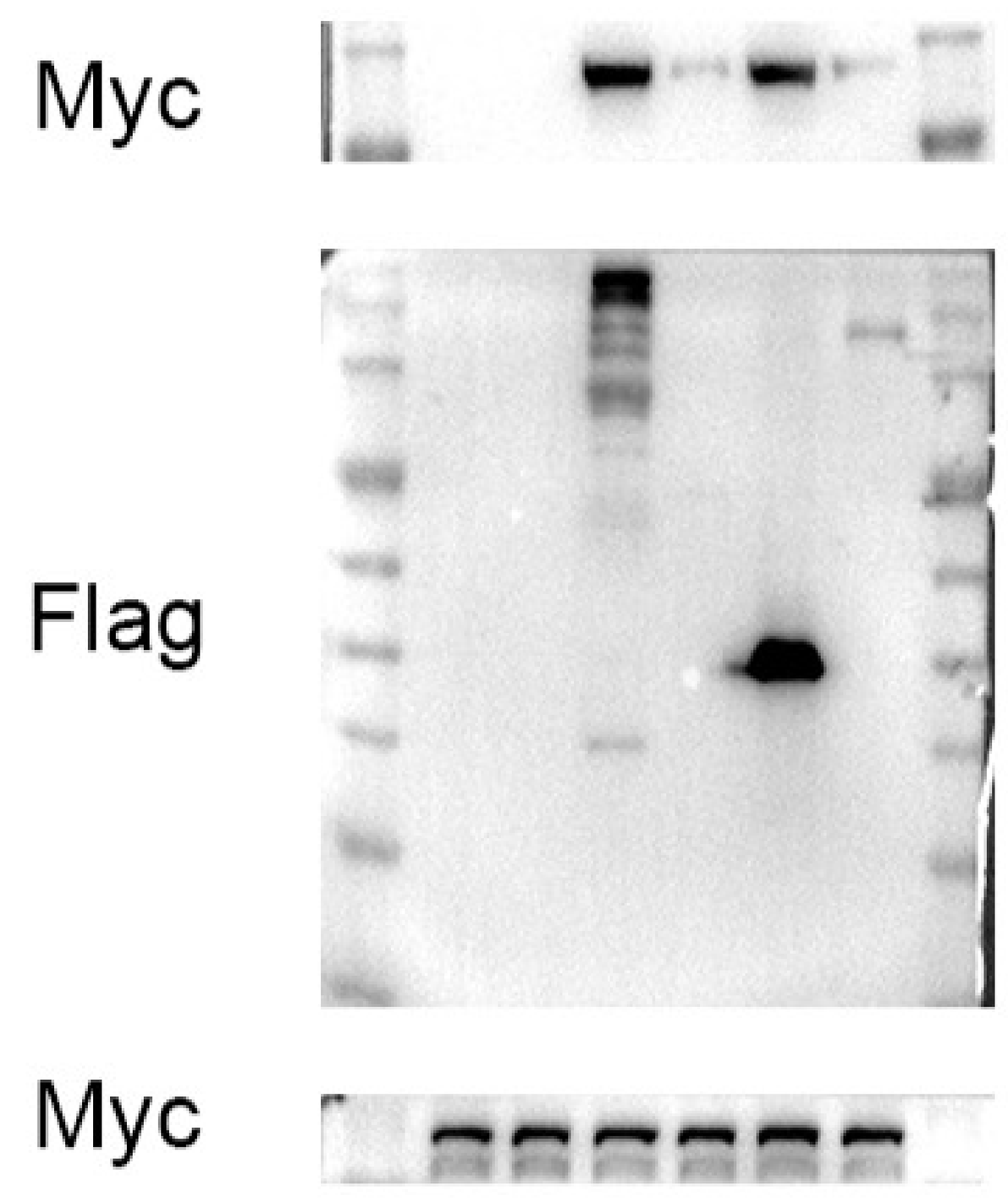

Figure 3G

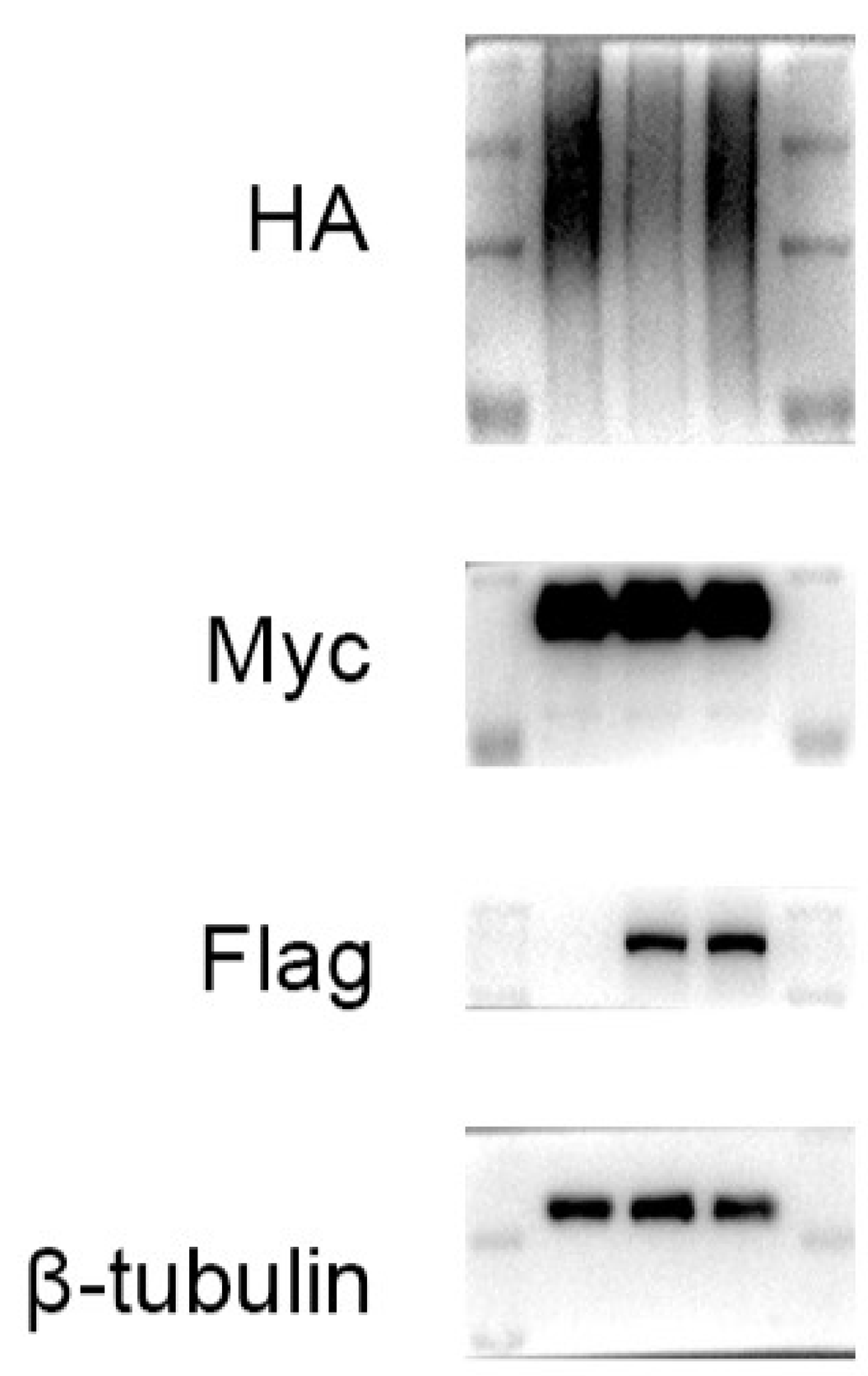

Figure 3H

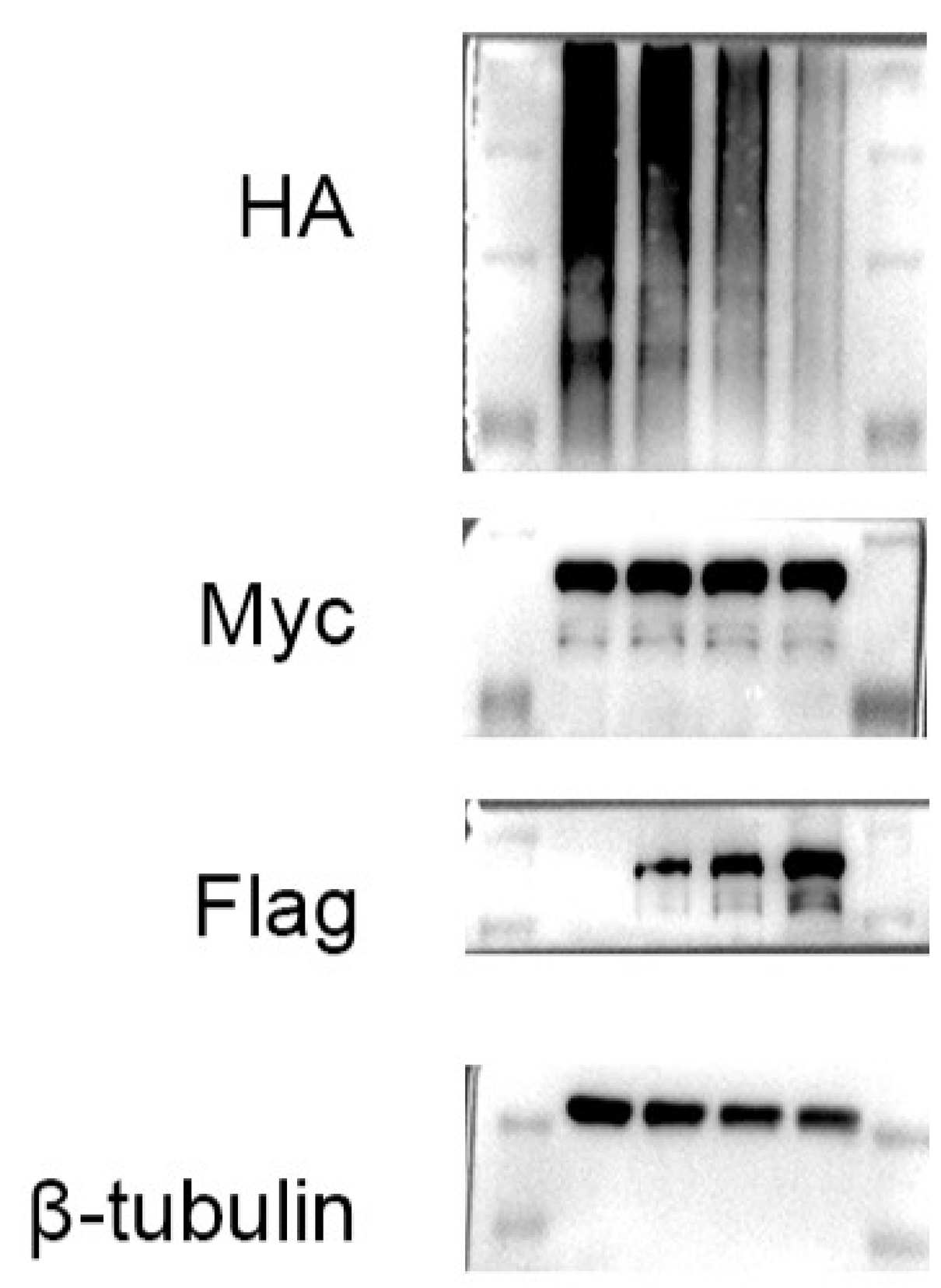

Figure 3I

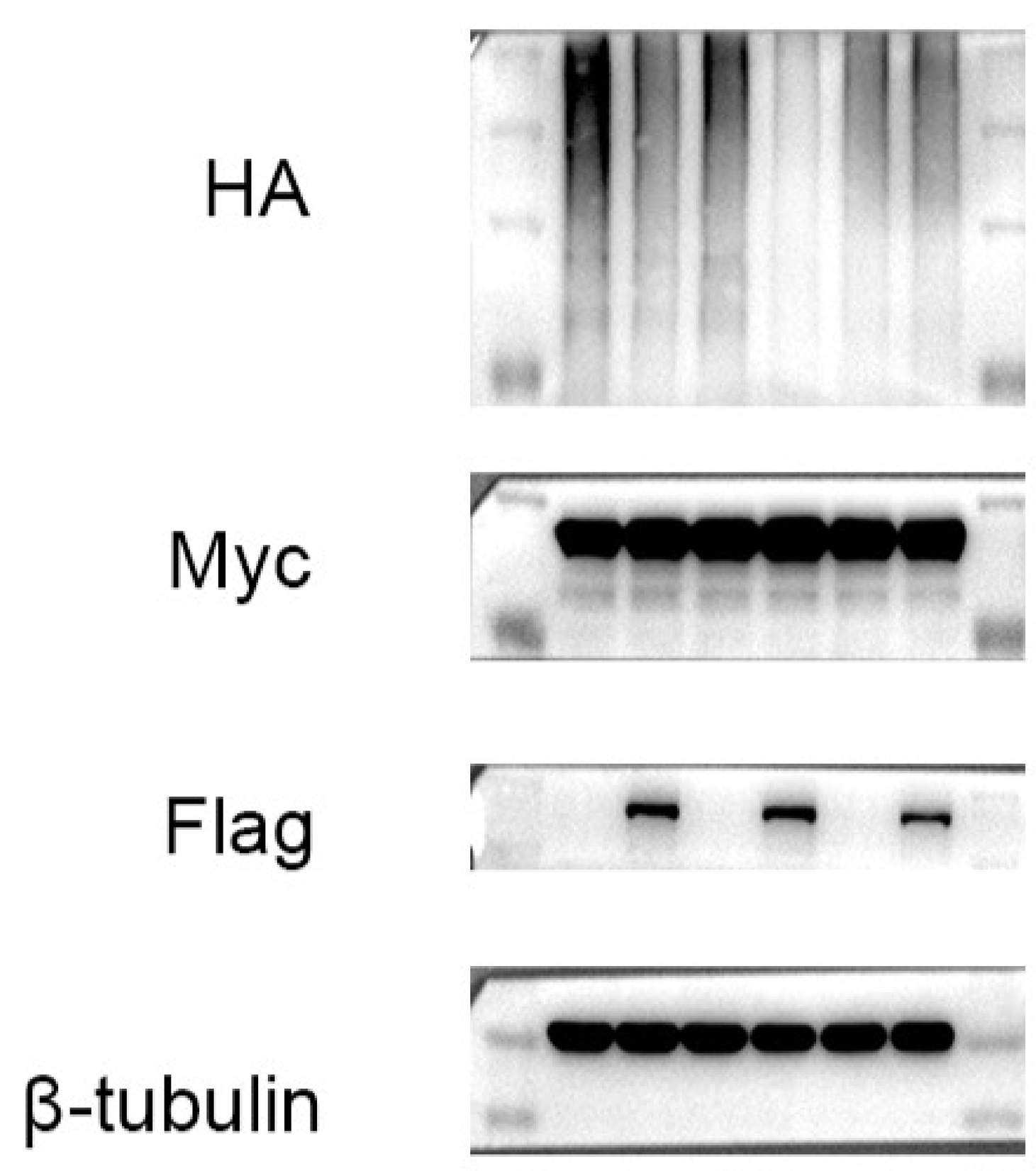

Figure 4A

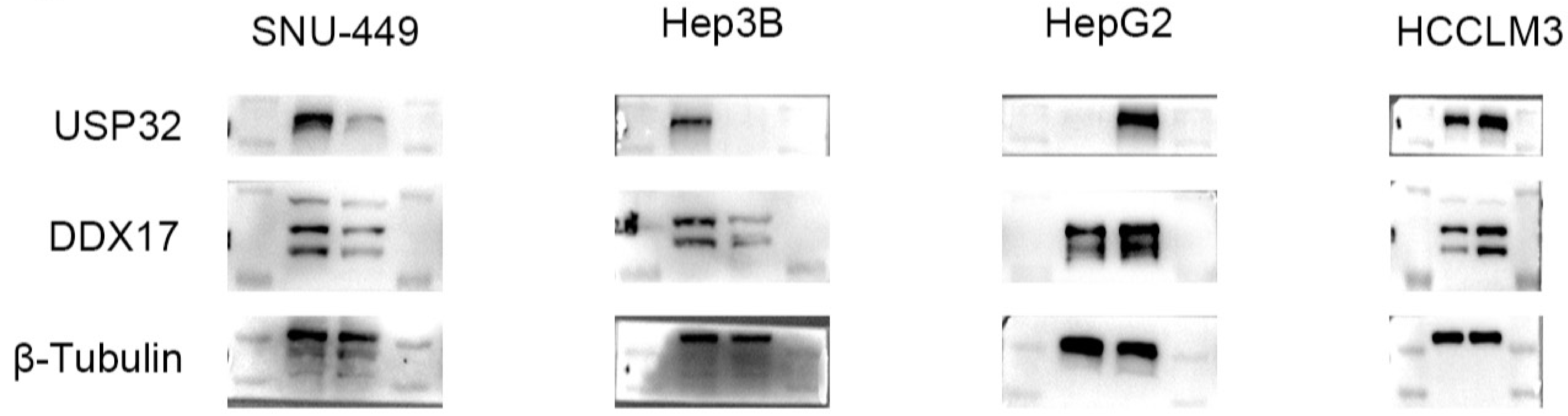

Figure 4C

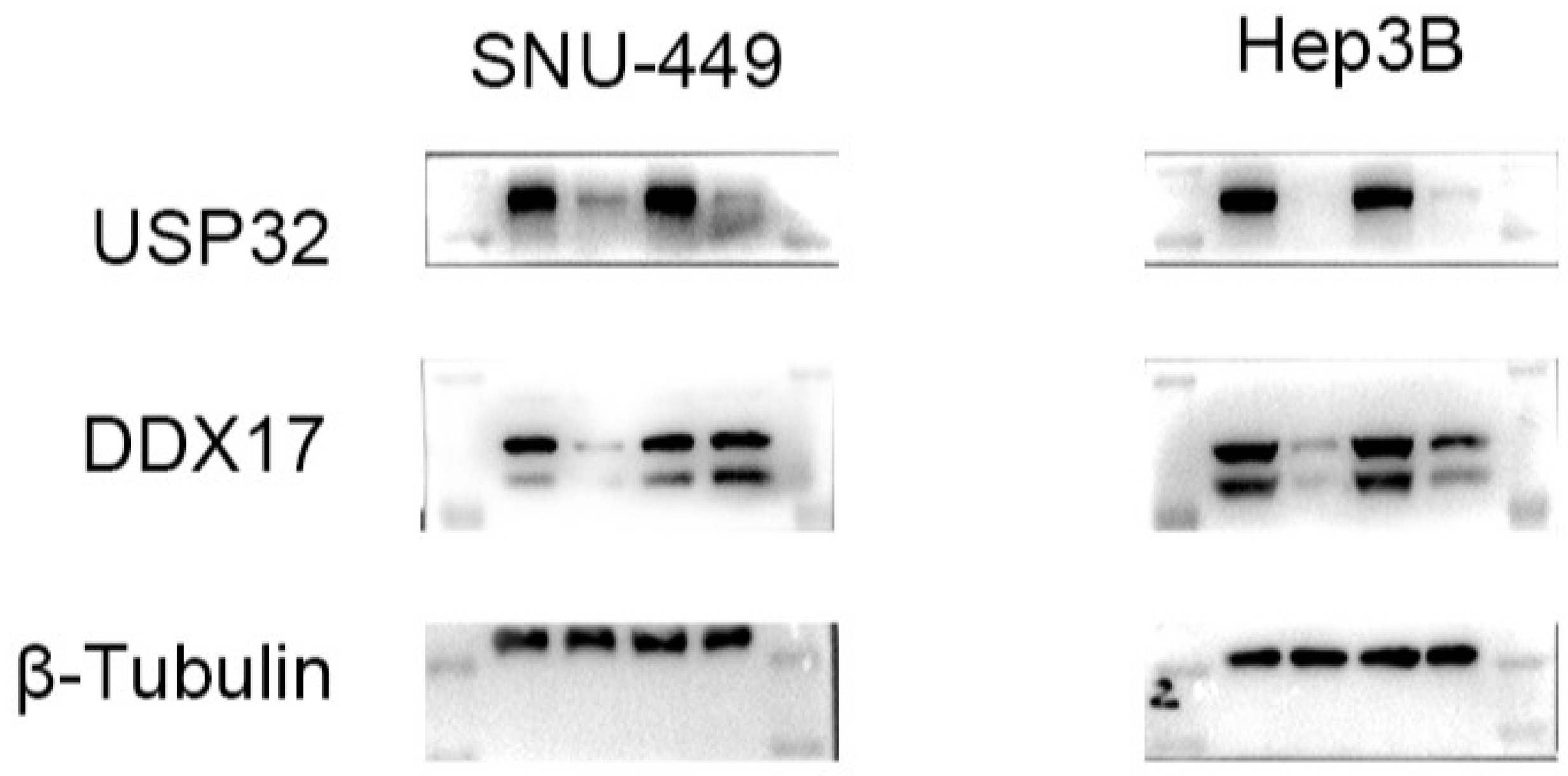

Figure 4D

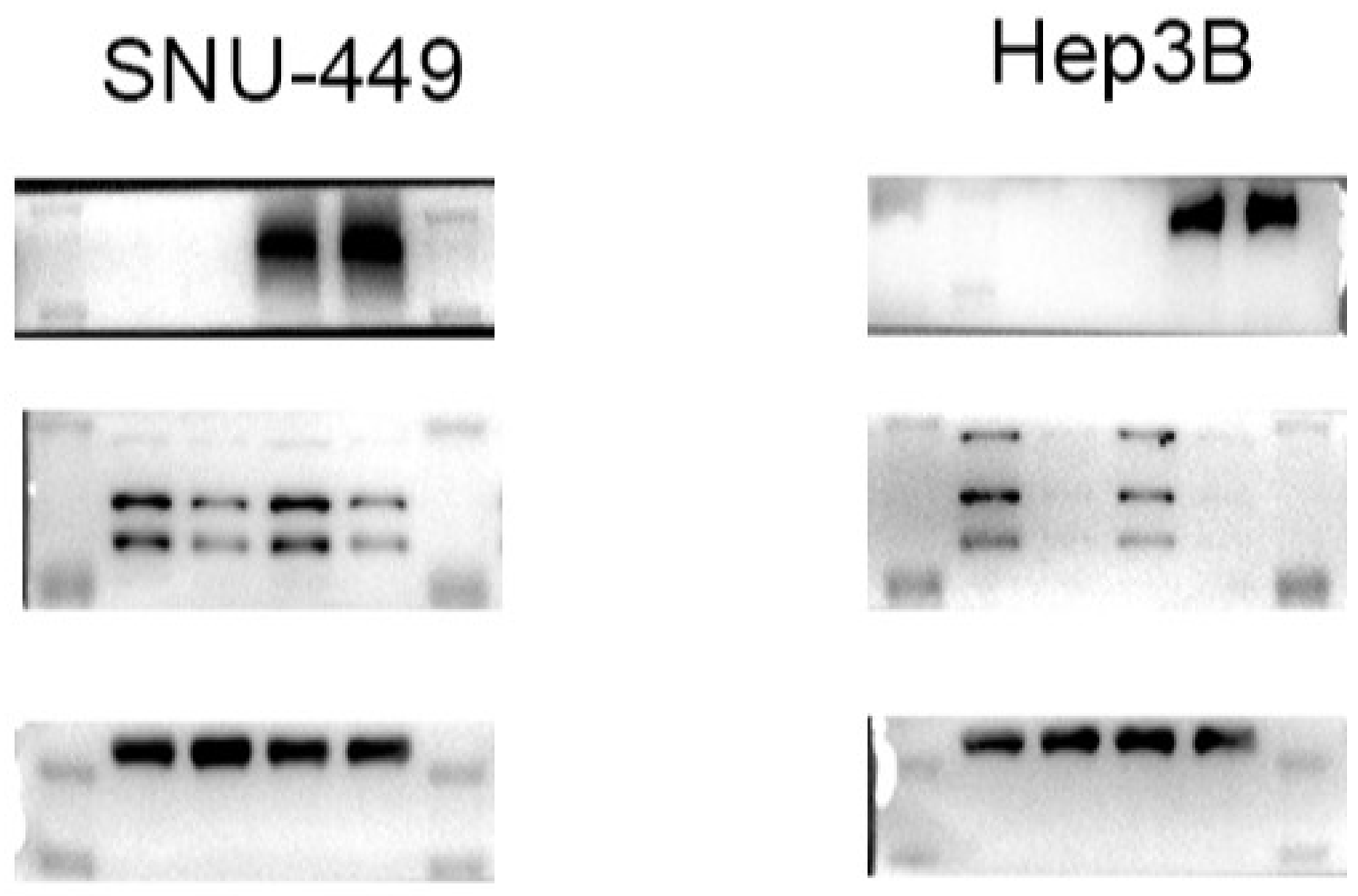

Figure 4E

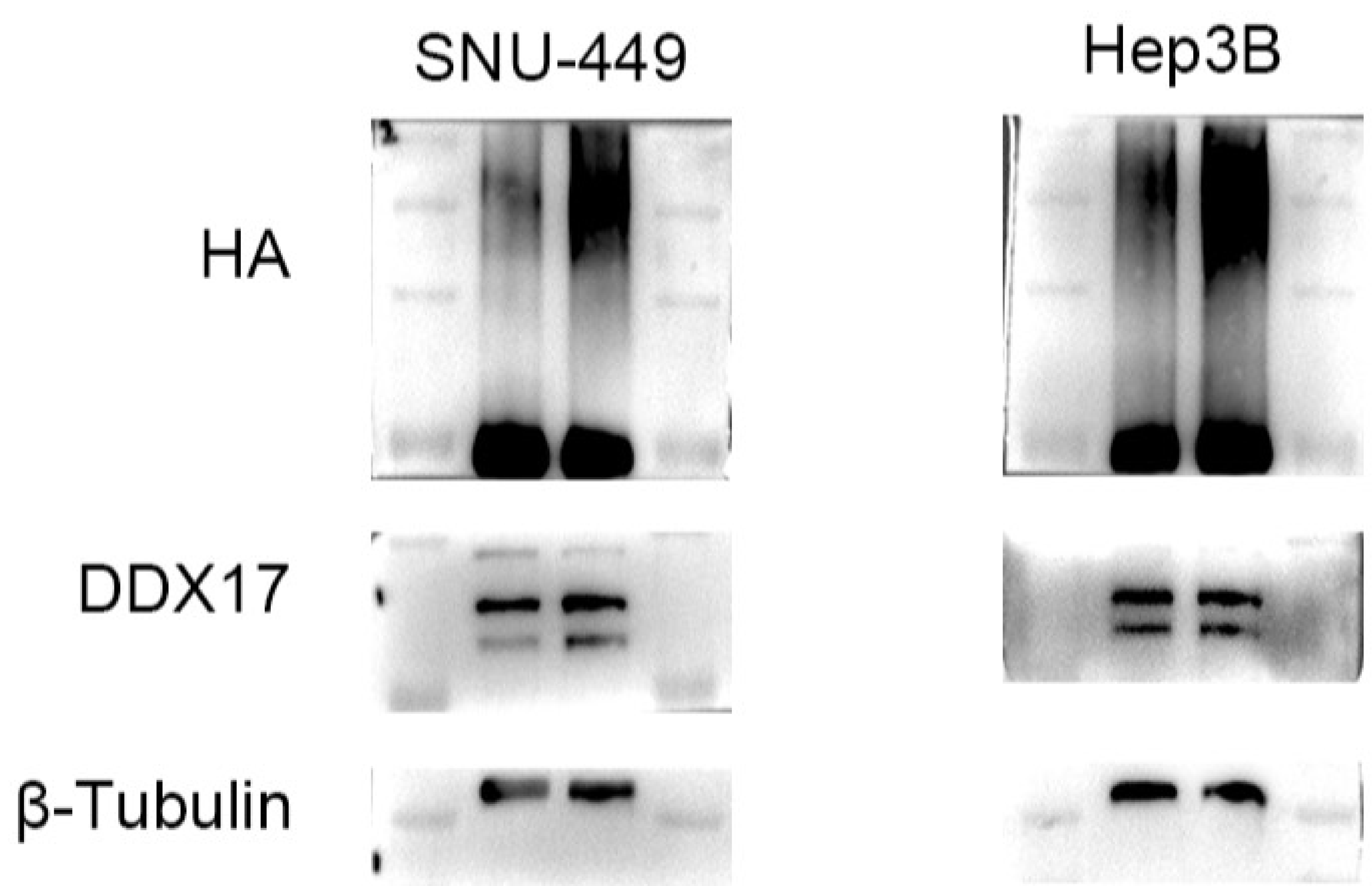

Figure 4F

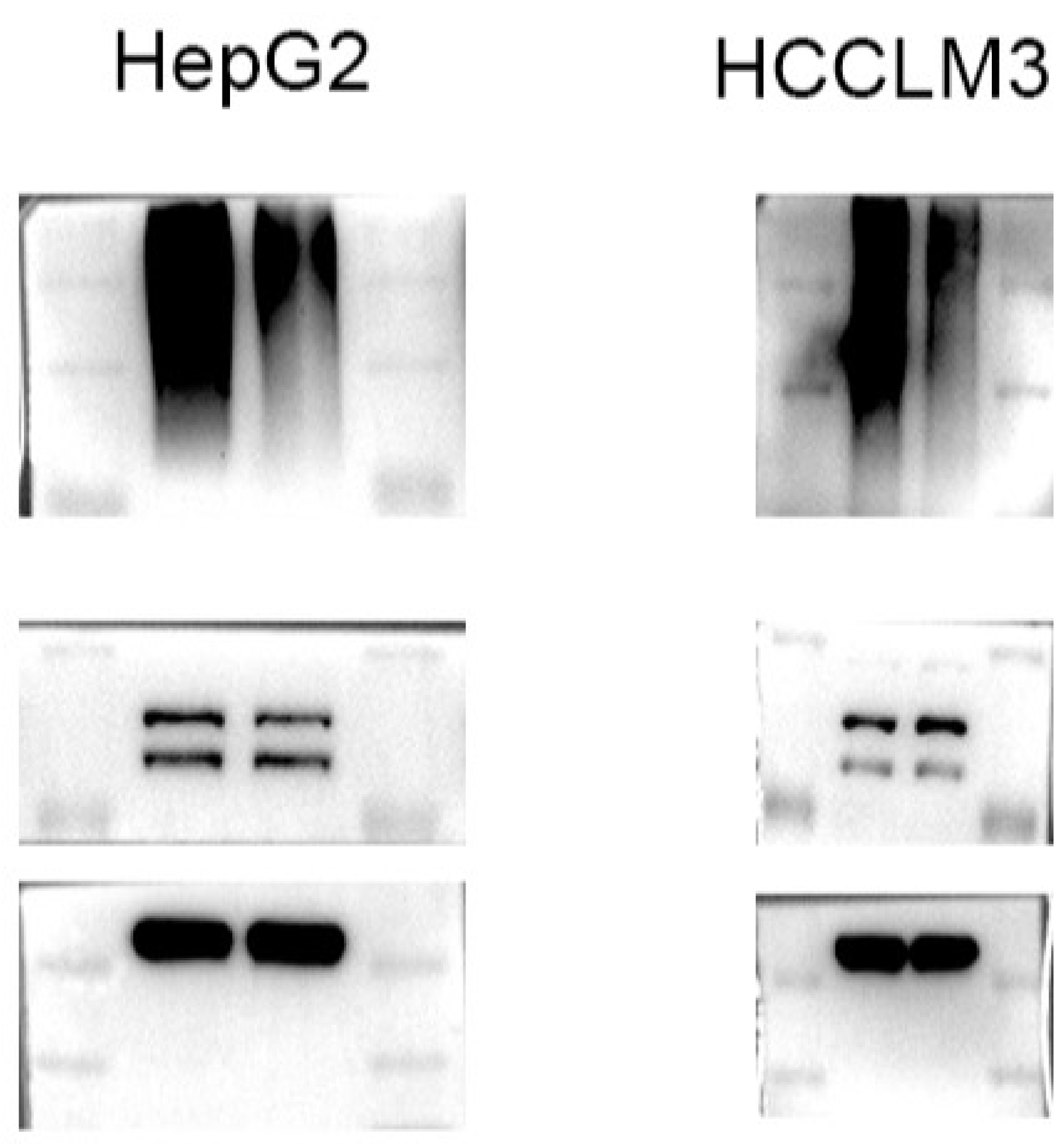

Figure 4G

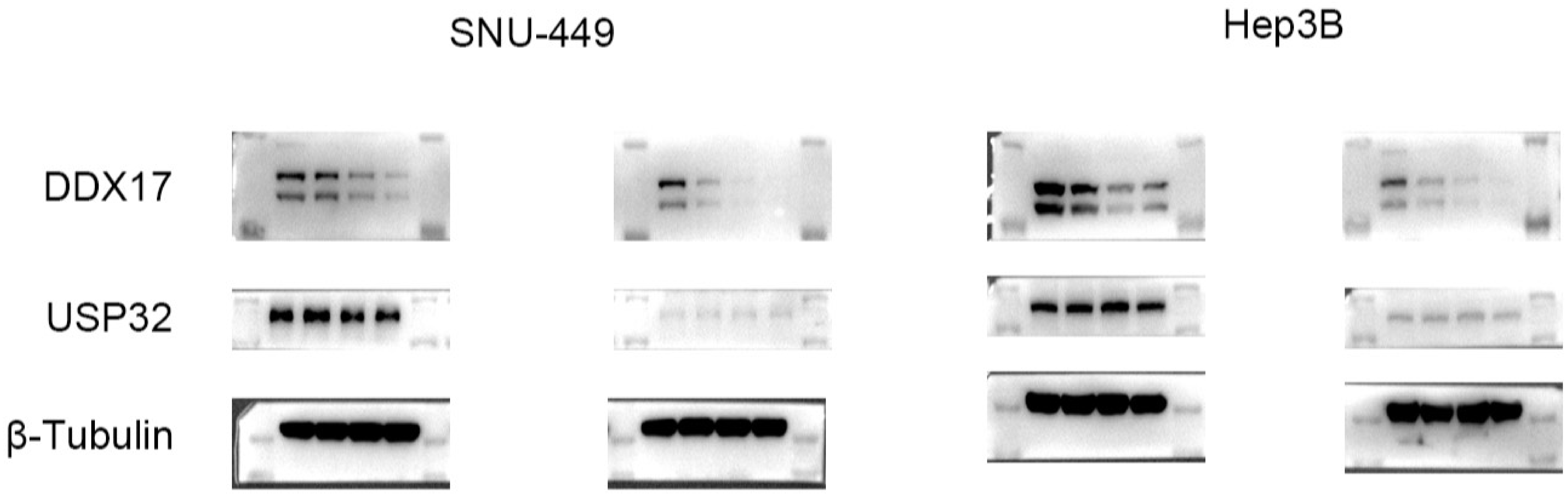

Figure 4H

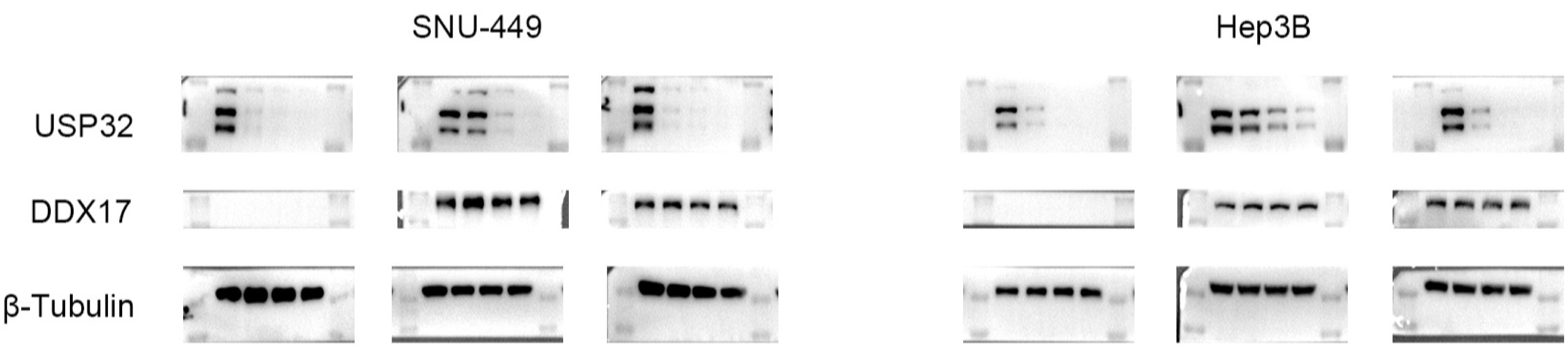

Figure 5H

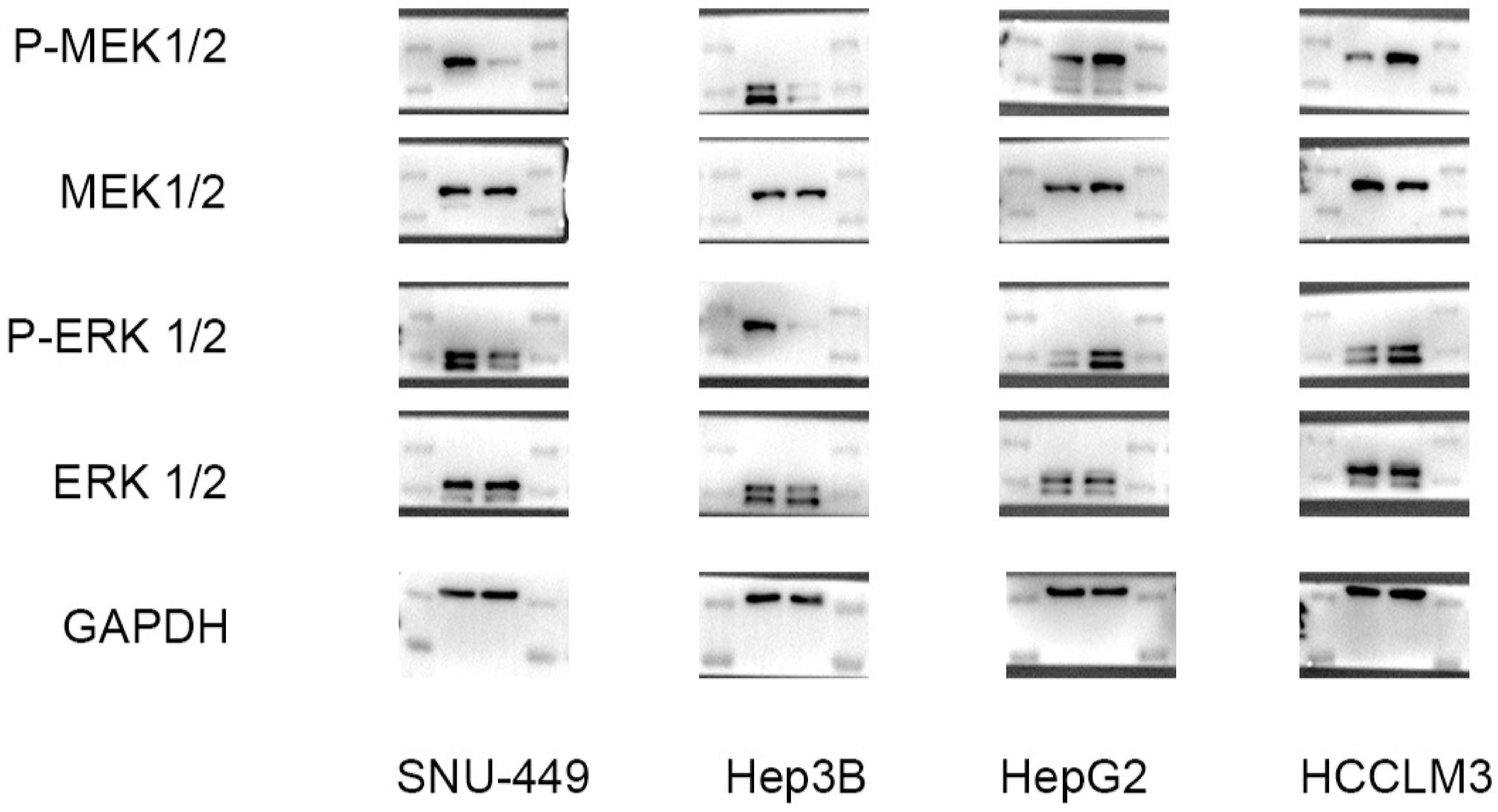

Figure 5I

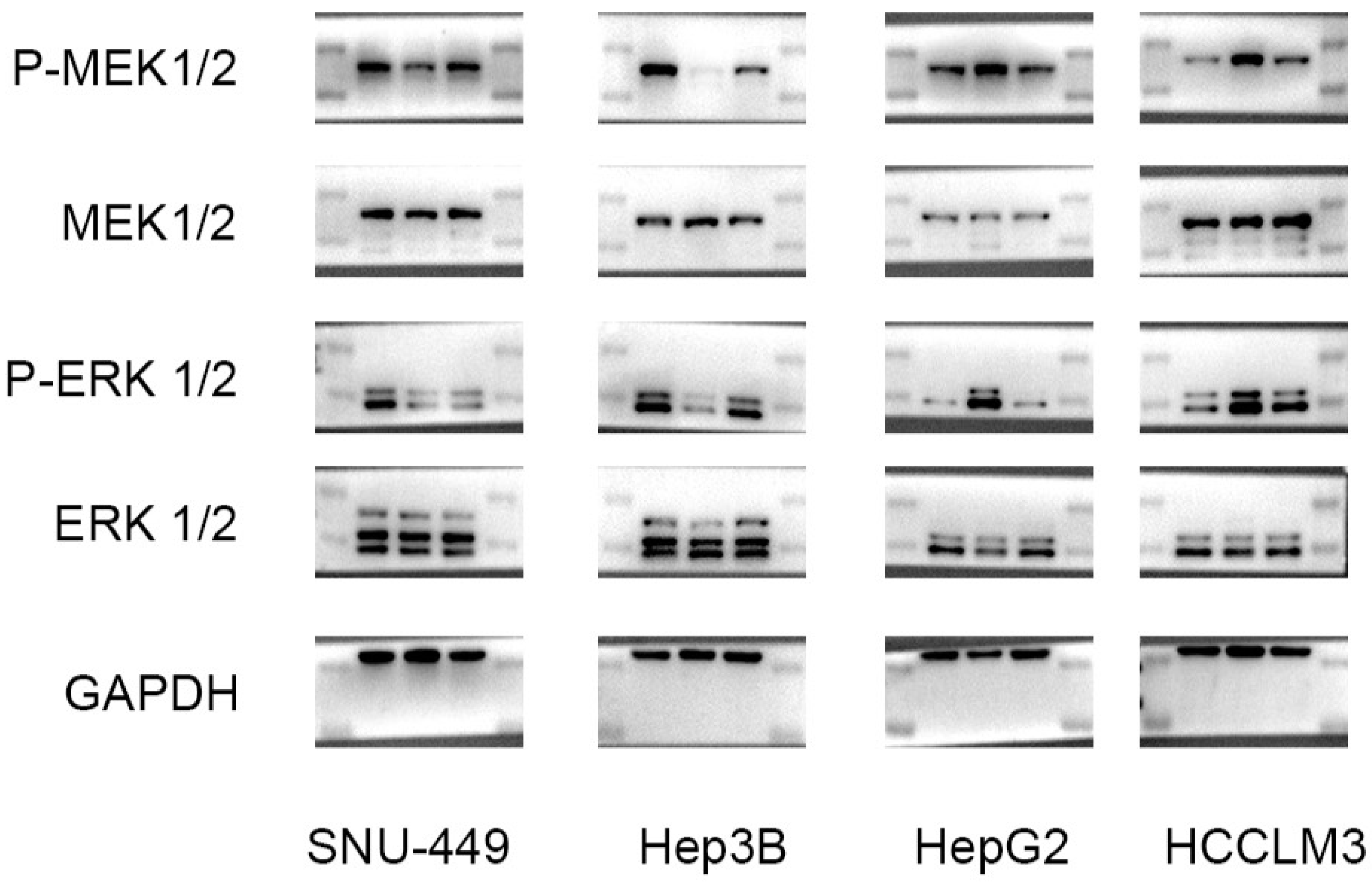

Figure 5J

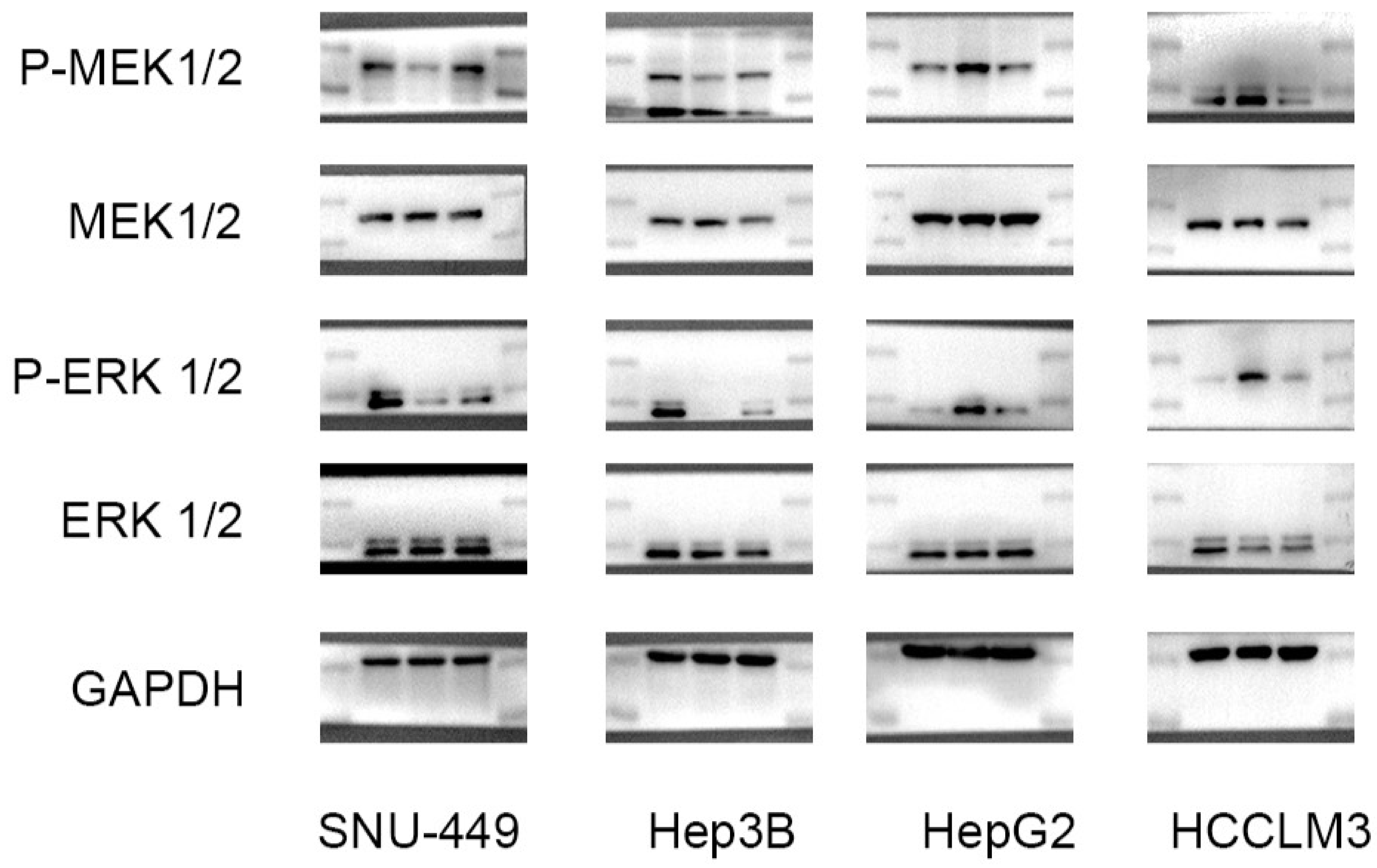

Figure 7C

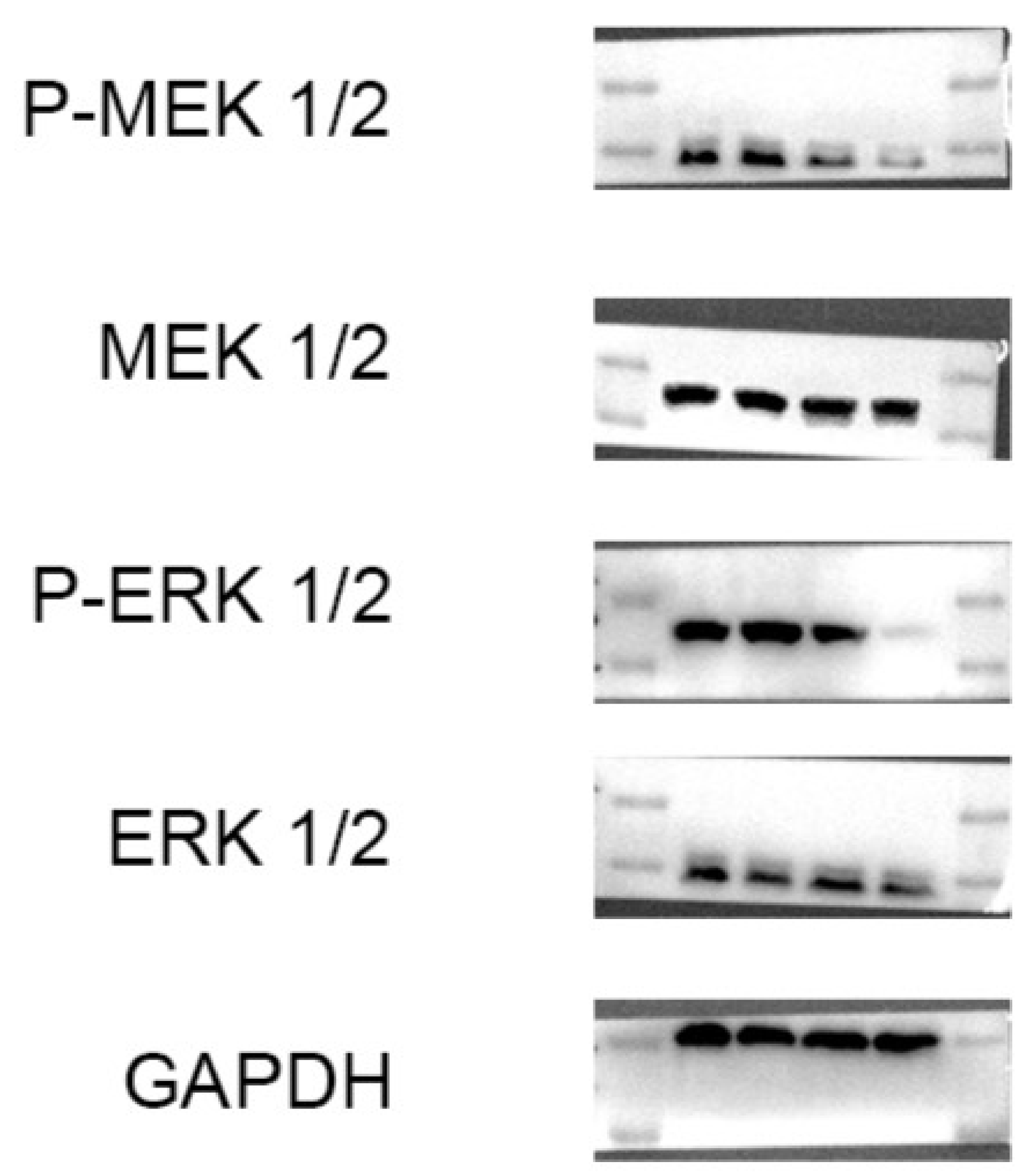

Figure 7D

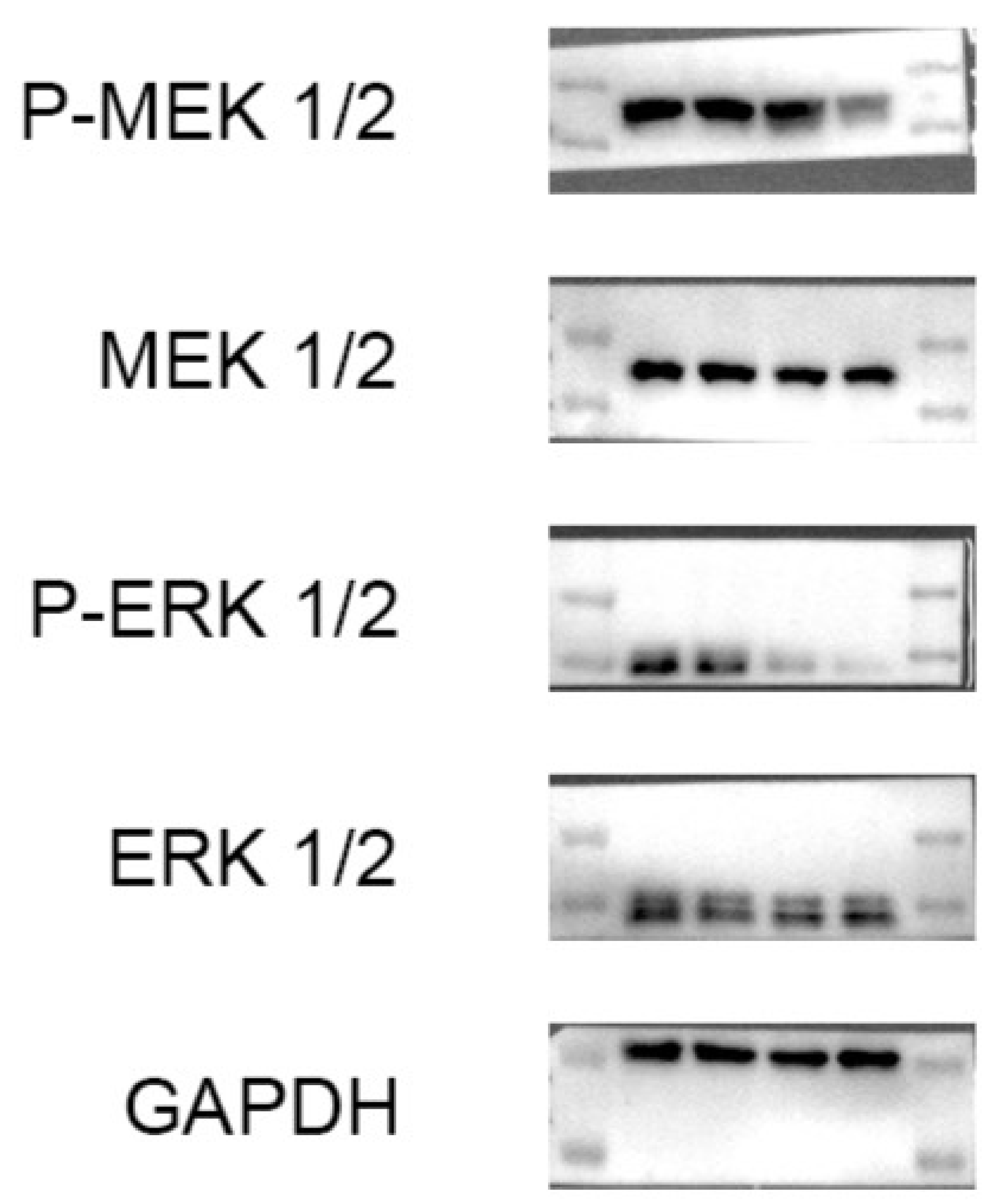

Figure S1

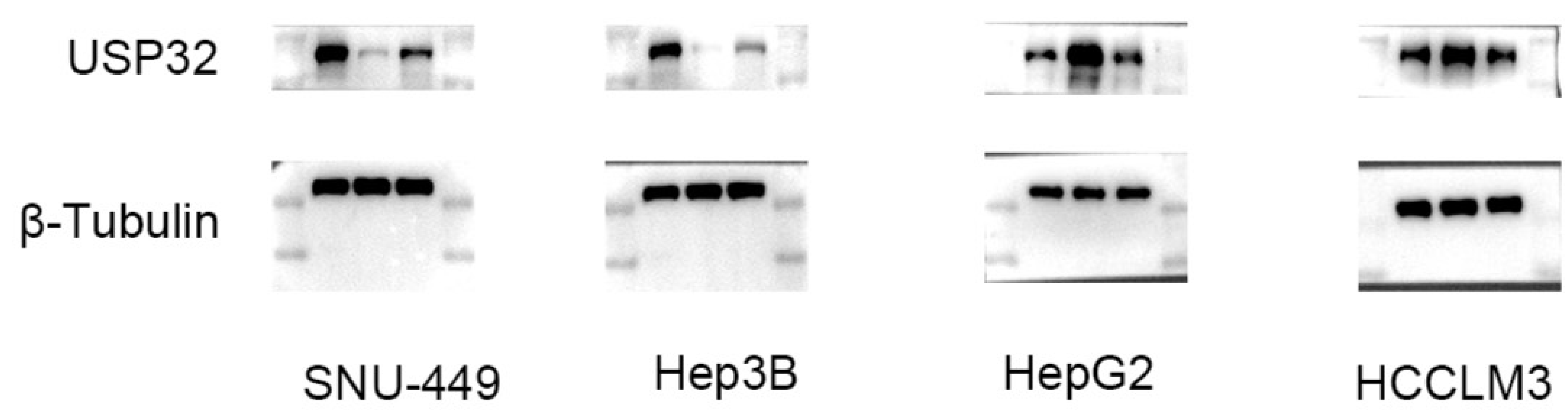

Figure S3

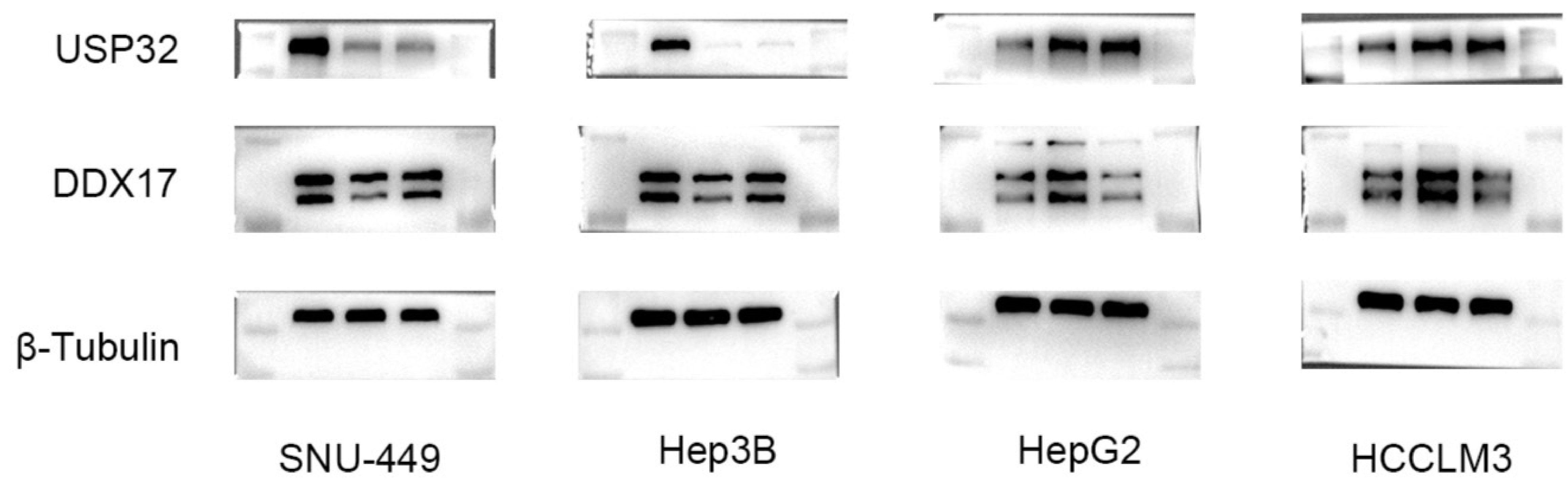

Supplement: Supplementary file 1 [file 41419_2026_9040_MOESM2_ESM.pdf]
