# Supplementary material for: Deubiquitinase USP32 stabilizes DDX17 to promote tumor progression and lenvatinib resistance in hepatocellular carcinoma via the miR-3163/MAPK pathway
Source: Cell Death Dis. 2026 Jun 25;17(1):789. doi: 10.1038/s41419-026-09040-1 (PMC13558724; doi:10.1038/s41419-026-09040-1)
Supplement: Supplementary file 2 [file 41419_2026_9040_MOESM1_ESM.docx]

**Supplementary information**

**Supplementary Figures**

**Supplementary Figure 1**

**Supplementary Figure 1. USP32 enhanced HCC cell proliferation. A** Western blot analysis validating simultaneous transfection of siRNA targeting USP32 (knockdown) and USP32-overexpressing plasmids in four HCC cell lines (SNU-449, Hep3B, HepG2, HCCLM3). **B** Colony formation assays demonstrating USP32-dependent promotion of HCC proliferation. **C** CCK-8 assays confirming that USP32 enhances the proliferative capacity of HCC cells. Statistical analysis, one-way ANOVA followed by Tukey’s comparisons test for (B), two-way ANOVA followed by Tukey’s comparisons test for (C). * *P* < 0.05, ** *P* < 0.01, *** *P* < 0.001, **** *P* < 0.0001.

**Supplementary Figure 2**

**Supplementary Figure 2. USP32 enhanced HCC cell migration and lenvatinib resistance. A, B** Scratch wound healing assays demonstrating USP32-dependent enhancement of HCC cell migration. **C, D** Transwell migration assays confirming the pro-migratory role of USP32 in HCC. **E, F** CCK-8 assays following treatment with a lenvatinib concentration gradient, showing that USP32 potentiates resistance to lenvatinib in HCC cells (E: *P*_1_: si-Ctrl + Vector vs si-USP32#2 + Vector and *P*_2_ : si-USP32#2 + Vector *vs* si-USP32#2 + OE-USP32; F : *P*_1_: Vector + si-Ctrl vs OE-USP32#2 + si-Ctrl and *P*_2_ : OE-USP32#2 + si-Ctrl *vs* OE-USP32 + si-USP32#2). Statistical analysis, one-way ANOVA followed by Tukey’s comparisons test for (C) and (D), two-way ANOVA followed by Tukey’s comparisons test for (E) and (F). * *P* < 0.05, ** *P* < 0.01, *** *P* < 0.001, **** *P* < 0.0001.

**Supplementary Figure 3**

**Supplementary Figure 3. USP32** **promotes HCC cell proliferation through DDX17-dependent manner. A** Immunoblot validation of USP32 knockdown and DDX17 overexpression in SNU-449/Hep3B cells, and USP32 overexpression with DDX17 knockdown in HepG2/HCCLM3 cells. **B** Colony formation assays demonstrating that DDX17 overexpression rescues proliferation defects caused by USP32 knockdown in SNU-449/Hep3B cells, while DDX17 knockdown reverses USP32-driven proliferation enhancement in HepG2/HCCLM3 cells. **C** CCK-8 assays confirm these reciprocal rescue effects on cell proliferation. Statistical analysis, one-way ANOVA followed by Tukey’s comparisons test for (B), two-way ANOVA followed by Tukey’s comparisons test for (C). * *P* < 0.05, ** *P* < 0.01, *** *P* < 0.001, **** *P* < 0.0001.

**Supplementary Figure 4**

**Supplementary Figure 4. USP32-DDX17 axis promotes HCC cell migration and lenvatinib resistance. A** Scratch wound healing assay demonstrating that DDX17 overexpression rescues impaired migration caused by USP32 knockdown in SNU-449 and Hep3B cells. **B** Forced USP32 expression enhances migratory capacity in Hep3B and HCCLM3 cells, which is reversed by DDX17 knockdown. **C** Transwell migration assays confirm that DDX17 overexpression restores migration defects induced by USP32 knockdown in SNU-449 and Hep3B cells. **D** USP32-driven migration enhancement in Hep3B and HCCLM3 cells is abrogated upon DDX17 silencing. **E** Dose-dependent lenvatinib treatment assays reveal that DDX17 overexpression re-sensitizes USP32-knockdown SNU-449 and Hep3B cells to lenvatinib (E: *P*_1_: si-Ctrl + Vector vs si-USP32#2 + Vector and *P*_2_ : si-USP32#2 + Vector *vs* si-USP32#2 + OE-DDX17) **F** DDX17 knockdown reverses USP32-mediated lenvatinib resistance in Hep3B and HCCLM3 cells (*P*_1_: Vector + si-Ctrl *vs* OE-USP32#2 + si-Ctrl and *P*_2_ : OE-USP32#2 + si-Ctrl *vs* OE-USP32 + si-DDX17#1). Statistical analysis, one-way ANOVA followed by Tukey’s comparisons test for (C) and (D), two-way ANOVA followed by Tukey’s comparisons test for (E) and (F). * *P* < 0.05, ** *P* < 0.01, *** *P* < 0.001, **** *P* < 0.0001.

**Supplementary Figure 5**

**Supplementary Figure 5. Dual inhibition of MAPK pathway in combination with lenvatinib synergistically overcomes drug resistance *in vivo* despite transient systemic toxicity. A, B** Baseline body weight (E) and tumor volume (F) in the transplanted model before treatment initiation. **C, D** Body weight changes in mice from induced (C) and transplanted (D) models across treatment groups. Transient weight loss was observed in combination groups. **E** In the induced lenvatinib-resistant HCC model, combination therapy (lenvatinib + selumetinib) achieved the most significant reduction in subcutaneous tumor volume. **F** Similarly, in the transplanted lenvatinib-resistant HCC model, dual therapy showed superior tumor suppression. Statistical analysis, one-way ANOVA followed by Tukey’s comparisons test for (A), (B), (E), and (F). ns not significant, * *P* < 0.05, ** *P* < 0.01, *** *P* < 0.001, **** *P* < 0.0001.

**Supplementary Figure 6**

**Supplementary Figure 6.** Schematic diagram depicting the proposed molecular mechanism.

**Supplementary Table 1**

| **siRNA** | **sequences** |
| --- | --- |
| si-USP32#1 | GAAGGACAACCGCACTGAT |
| si-USP32#2 | ACGACAGTATGGGCTATCA |
| **shRNA** |  |
| sh-USP32#1 | CCGGGGGTTGAACTGAGAGACATGGCTCGAGCCATGTCTCTCAGTTCAACCCTTTTTT |
| sh-USP32#2 | CCGGGCAGGACACAACTGGTTTATCCTCGAGGATAAACCAGTTGTGTCCTGCTTTTTT |
| sh-USP32#3 | CCGGGCTTTGCTCGACAACATAACACTCGAGTGTTATGTTGTCGAGCAAAGCTTTTTT |
| sh-USP32#4 | CCGGGCCATTAGTAACTCAAGAACCCTCGAGGGTTCTTGAGTTACTAATGGCTTTTTT |

| **siRNA** | **Sequences** |
| --- | --- |
| si-DDX17#1 | AGAGGAUUUCCUUCGUGAUTTAUCACGAAGGAAAUCCUCUTT |
| si-DDX17#2 | CCACAAGUUGAUCCAACUATTUAGUUGGAUCAACUUGUGGTT |
| **shRNA** | **Sequences** |
| sh-USP32#1 | CCGGGGGTTGAACTGAGAGACATGGCTCGAGCCATGTCTCTCAGTTCAACCCTTTTTT |
| sh-USP32#2 | CCGGGCAGGACACAACTGGTTTATCCTCGAGGATAAACCAGTTGTGTCCTGCTTTTTT |
| sh-USP32#3 | CCGGGCTTTGCTCGACAACATAACACTCGAGTGTTATGTTGTCGAGCAAAGCTTTTTT |
| sh-USP32#3 | CCGGGCCATTAGTAACTCAAGAACCCTCGAGGGTTCTTGAGTTACTAATGGCTTTTTT |

| **Primers** | **Sequences** |
| --- | --- |
| GAPDH-F | ACGGGAAGCTTGTCATCAAT |
| GAPDH-R | TGGACTCCACGACGTACTCA |
| USP32-F | TGGCAATGGCTACAGCAATGGTC |
| USP32-R | AGGCAGAGTCGGTGTCAATTTCATC |
| DDX17-F | TGGGCTAGATGTGGAAGATGTCAAG |
| DDX17-R | TGTTGGTGCTACGGGCTGTTC |
| hsa-miR-885-5p-F | GCTCCATTACACTACCCTGCCTCT |
| hsa-miR-3163-F | CCGGCTATAAAATGAGGGCAGTAAGAC |
| hsa-miR-3149-F | CGCGCCTTTGTATGGATATGTGTGTG |
| hsa-miR-885-3p-F | ATAAAGGCAGCGGGGTGTAGTGGATA |
| hsa-miR-675-3p-F | CTGTATGCCCTCACCGCTCA |
| hsa-miR-4798-5p-F | CGCGGTTCGGTATACTTTGTGAATTGG |
| hsa-miR-3616-5p-F | CCGCATGAAGTGCACTCATGATATGT |
| hsa-miR-4746-5p-F | ATTCCGGTCCCAGGAGAACCT |
| hsa-miR-675-5p-F | ATTAATTTGGTGCGGAGAGGGCC |
